# Mapping one health surveillance and networks for vector-borne disease prevention and control in Europe – A scoping review

**DOI:** 10.1016/j.onehlt.2026.101545

**Published:** 2026-08-18

**Authors:** Godfred Amankwaa, Emmanuel S. Tomude, Hermann Kam, Bethan V. Purse, Jonathan Rushton, Mara Rocchi, Kayleigh Hansford, Jolyon M. Medlock, Nicholas Johnson, Festus A. Asaaga

**Affiliations:** a UK Centre for Ecology & Hydrology, Maclean Building, Crowmarsh Gifford, Wallingford OX10 8BB, United Kingdom; b School of Geography, University of Nottingham, University Park NG7 2RD, UK; c Roslin Institute, University of Edinburgh, Easter Bush Campus, Midlothian, EH25 9RG, UK; d Moredun Research Institute, Pentlands Science Park, Penicuke, EH26 OPZ, UK; e Medical Entomology & Zoonoses Ecology, UK Health Security Agency, Porton Down, UK; f Vector Borne Diseases, Animal and Plant Health Agency Weybridge, Surrey KT17 3NB, UK

**Keywords:** One health surveillance, Vector borne disease, Europe, Cross-sectoral integration

## Abstract

The increasing vector-borne disease (re)emergence and geographic expansion, driven by environmental change, has been accompanied by growing calls for integrated or One Health surveillance approaches across the human, animal, and environmental health sectors. However, despite widespread advocacy and policy implementation, assessment of the extent to which existing systems achieve cross-sectoral integration remains unclear. There also remains limited evidence regarding existing surveillance capacities and the priorities of key actors within the surveillance ecosystem for transitioning to effectively integrated One Health (OH) surveillance systems, particularly in the context of vector-borne diseases. In this paper, we present the first evidence synthesis and mapping of the One Health Vector Borne Disease (VBD) surveillance systems landscape across Europe and the UK. We conducted targeted searches across Web of Science, PubMed, Google Scholar, and Cochrane Library supplemented by grey literature and institutional searches across national surveillance organisations, which led to retrieval of 25 peer-reviewed papers and 72 distinct VBD surveillance systems. Of the 25 papers, overall profiling showed the OH VBD surveillance literature to be recent and predominantly quantitative, dominated by epidemiological and public health disciplines and concentrated in Northern/Western Europe. Also, there is limited use of evaluation frameworks for surveillance operationalisation and impact assessments across the reviewed literature. Analysis of the 72 surveillance systems showed that despite One Health rhetoric and government funding of over 90% of systems, there was persistent sectoral fragmentation, with systems skewed toward human health and animal health domains. Whilst institutional coordination existed in approximately 75% of systems, operational integration such as coordinated sampling and risk communication remained limited, and inclusion of the environmental health sector was underrepresented. We end by laying out opportunities for future research, with particular emphasis on the evaluation of effectiveness of surveillance and integration of social science and interdisciplinary perspectives. We also highlight opportunities for investing and funding the co-development and cross sectoral operationalisation for VBD surveillance and control.

## 1 Background

The increasing emergence of infectious diseases globally is precipitating wide-ranging societal, economic, health and welfare impacts and continues to gain prominence across global health security and climate emergency agendas. Available statistics indicate that zoonoses, diseases transmitted from animals to people, account for 2.5 billion cases of human illness and 2.7 million human deaths worldwide annually [1], [2], [3]). Vector-borne diseases are also showing particularly dynamic geographical shifts, with diseases such as dengue, chikungunya, Lyme disease (LD), tick-borne encephalitis (TBE), and Crimean-Congo Haemorrhagic Fever (CCHF) expanding into new areas [4], [5], [6], [7]. These shifts are driven by major factors including climate change, habitat modification, urbanisation, and pathogen and vector evolution at the human-animal-ecosystem interface [4], [5], [8], [9]. In Europe, for instance, tick-borne and mosquito-borne diseases are of particular concern due to their sensitivity to ecological changes and complex host-pathogen transmission dynamics [8], [10]. It is estimated that reported cases of Lyme disease in the UK have increased ten-fold since 2000 [11], partly linked to an expanding distribution of its main tick vector *Ixodes ricinus* [8], [12], but also due to greater awareness of the disease [13]. Similarly, Crimean-Congo Haemorrhagic Fever (CCHF), caused by a WHO priority pathogen with epidemic potential, is expanding westward across Europe [14], [15]. Tick-borne encephalitis virus (TBEV) has also demonstrated significant northward and westward expansion, establishing new endemic foci in previously unaffected European regions [16], [17], [18]. These geographical shifts underscore the need for adaptive surveillance approaches [19].

In response to these emerging and endemic threats, international health organisations and national agencies have increasingly advocated for One Health surveillance systems spanning the human-animal-ecosystem interface [20], [21]. For instance, institutions such as the World Health Organisation (WHO), the European Food Safety Authority (EFSA), and the European Centre for Disease Prevention and Control (ECDC) have championed coordinated surveillance approaches that transcend traditional sectoral boundaries, with calls for data collection on disease outbreaks, trends, and emergence detection across multiple domains to inform evidence-based decision-making and enable rapid response to emerging health threats [22], [23], [24]. Within public health practice, considerable attention and resources have been devoted to surveillance of foodborne diseases and antimicrobial resistance, yielding notable successes in managing these challenges across environmental, species, and sectors [25], [26]. Similarly, the One Health discourse has been accompanied by a burgeoning body of research exploring (integrated) surveillance frameworks, with growing evidence of the potential benefits such as improved early warning, cross-sectoral data sharing, cost effectiveness and more coordinated outbreak response and collaborative approaches that span traditional disciplinary and sectoral boundaries in different contexts [e.g. [27], [28], [29], [30], [31].

However, despite these advances and the increasing recognition of interconnected disease threats [32], [33], current routine surveillance systems continue to operate predominantly within individual sectoral silos. Whilst the One Health approach provides the conceptual platform for operationalising integrated surveillance infrastructure that bridges data generation gaps and facilitates information dissemination for decision-making [34], [35], confusion and uncertainty regarding practical application, outcomes, and impacts prevail, with limited conceptual clarity about implementation pathways [28], [36]. Most existing surveillance systems focus on known diseases and clinical syndromes within specific domains, with early detection and response capabilities often constrained by limited cross-sectoral coordination and information sharing [23], [37]. This fragmentation is particularly problematic for zoonotic and vector-borne diseases, where environmental, animal, and human health factors interact in complex ways that cannot be adequately captured through single-sector surveillance approaches.

Despite increased research and policy focus on integrated surveillance approaches [21], [23], [28], [38], there remains limited evidence regarding existing surveillance capacities, cross-sectoral interactions, and the priorities and capabilities of key actors within the surveillance ecosystem for transitioning to effectively integrated One Health surveillance systems particularly in the context of vector-borne diseases and beyond the (sub)national scale. Within the OH surveillance literature, much of the existing research has narrowly focussed on individual or specific VBD threats in localised case studies and populations, with limited generalisability at a broader regional scale [38], [39], [40]. This also calls for secondary studies to map the spectrum of surveillance initiatives and networks for different VBD threats on a pan-European scale. Contributing to this emerging and increasing scholarship, with the aim of identifying integration points, gaps, and opportunities, this paper is guided by three key questions: (1) What research evidence exists to inform One Health surveillance approaches for vector-borne diseases across research domains and what remains to be researched across Europe? (2) To what extent are existing surveillance and monitoring systems for prevention and control of vector-borne diseases cross-sectoral in design and function across Europe? and (3) Do surveillance and monitoring systems relevant to vector-borne diseases meet One Health attributes of integrated surveillance systems?

## 2 Methods

Following Preferred Reporting Items for Systematic Reviews and Meta-Analyses extension for Scoping Reviews (PRISMA-ScR) guidelines [41], [42], [43], we conducted a scoping review to identify and characterise past, ongoing, and planned surveillance initiatives, programmes, data sources, and exchange systems for vector-borne diseases, with particular focus on their implementation of One Health governance structures. In this paper, we define a surveillance system, initiative, or programme as any structured, ongoing effort to systematically collect, collate, analyse, interpret, and disseminate health-related data on a defined hazard or across multiple domains [28] for the purpose of informing prevention, control, or response action, encompassing databases, networks, dashboards, and applications that fulfil this function irrespective of technical form. Identified entities are further classified according to the typology applied in dimension (ii) of our data extraction framework (Supplementary Material 4): (1) surveillance systems, (2) disease control systems, (3) vector control programmes, and (4) other intervention systems.

### 2.1 Key literature and surveillance search strategy

To identify national and regional surveillance ecosystems, networks, and infrastructure for surveillance and control of vector-borne diseases in the UK and Europe, we conducted a scoping search in three related parts. First, we conducted systematic searches across six online bibliographic databases including Web of Science, PubMed, Google Scholar, Cochrane Library and Ovid (Medline). At the outset, we conducted a topic search in Cochrane Library database to identify any prior systematic reviews which yielded non results. Subsequently, systematic searches (see Table S1 in Supplementary Material 1) were conducted in Web of Science and PubMed to identify relevant peer-reviewed literature given their wide scope of interdisciplinary scientific publications, refined between October 2023 and September 2025. For Google Scholar, we inspected the first two pages of results and subsequently screened additional pages (500 hits in total) until no more relevant results were found. We limited searches by geographical focus on the UK (England, Wales, Scotland, and Northern Ireland) and Europe, language of publication (English), and searched all databases from 1st January 2000. The key search terms applied are presented in Table S1. Second, we undertook backward and forward citation tracing from articles identified through database searches. From this, we retrieved an additional 18 papers.

In the third stage, we conducted rapid textual mining of grey documents retrieved via targeted online searches of institutional websites (DEFRA, UKHSA, Public Health Scotland, APHA, other Europe wide sources etc). Using structured search strings combining “surveillance”, “monitoring”, and “vector-borne diseases” with country identifiers, we identified surveillance programmes and initiatives. Additional searches focused on European-level surveillance networks through ECDC, EFSA, and WHO European Region platforms. We sourced technical documentation including surveillance evaluation reports, and annual summaries from governmental repositories. Surveillance systems named or identified within our screened articles and final corpus papers formed the initial basis for Table 2, which was subsequently expanded through the grey literature and institutional searches described above.

### 2.2 Article selection

Search results from all databases were uploaded and imported into Sciwheel reference management software (https://sciwheel.com/?lg) where duplicates were removed. Here, the de-duplicated articles were screened by four independent reviewers (GA, EST, HK & FAA) who conducted title-abstract and full-text screening, using the inclusion and exclusion criteria (see Table 1). Any disagreements were resolved through team discussions.

**Table 1:** Inclusion and exclusion criteria.

**Inclusion**	**Exclusion**
• Study must describe a surveillance system, initiative, or programme for disease control.	• Paper/ study not focused on a surveillance system, initiative, or programme for disease control.
• The focus of the surveillance system, initiative, or disease control programme should be on vector-borne disease hazards.	• Not focused on vector borne diseases
• The surveillance system, early warning information, or forecasting systems or initiative must be operationalised in the UK/ EU or any of its devolved administrations.	• Surveillance system, early warning information, or forecasting systems or initiative not operationalised in the UK/ EU or any of its devolved administrations.
• Peer reviewed research papers	

### 2.3 Data extraction and analysis

To code reviewed papers, we developed a data extraction framework in KobolCollect Toolbox (see Supplementary Material 4) comprising nine key dimensions: (i) study characteristics, (ii) surveillance system or disease control programme description, (iii) design of surveillance or control programme, (iv) content (focal diseases, pathogens), (v) regulatory environment, (vi) governance and coordination of the surveillance system, (vii) collaborative typology, (viii) funding and sustainability, and (ix) evaluation of One Health implementation in surveillance initiatives. Data was extracted by two reviewers (GA & FAA) and exported to a Microsoft Excel spreadsheet for analysis. The coding scheme was iteratively applied, first by one author and then through co-coding of the first five papers by two authors (GA, FAA), leading to further revisions and finalisation of categories and codes. Following standard scoping review practice, a formal risk of bias assessment was not conducted [44].

Also to meet the second and third objectives of the study, the selected surveillance systems were assessed according to predetermined variables in the coding schema (substantive dimensions ii-ix), allowing description of the content, design, regulatory typology, organisation, functioning, surveillance context, health hazards and domains under surveillance, and collaborative typology including types of collaboration and underlying mechanisms. Variables related to One Health evaluation were slightly refined during the information collection process to capture the different dimensions and characteristics of the systems. One Health-ness was assessed using dimension (ix) of the coding framework (Supplementary Material 4), which scores the extent to which policy (sectoral mandates and governance), institutional (coordination mechanisms and participation), and operational (data exchange, joint analysis, resource sharing, protocol harmonisation, and coordinated sampling) dimensions were considered, scored independently so that a system could show integration at one level without necessarily showing it at another. Subsequently, deductive content and thematic analysis of the selected papers and identified systems was undertaken.

## 3 Results and synthesis

Here, we present the thematic mapping from the systematic scoping review, organised into profiling the state of the literature to date, characterising the current landscape of surveillance initiatives, their operational characteristics, geographical coverage and representation, and the extent of cross-sectoral integration.

### 3.1 Characteristics of selected studies and literature landscape

#### 3.1.1 Profile of literature on VBD surveillance systems

**Fig. 1:**
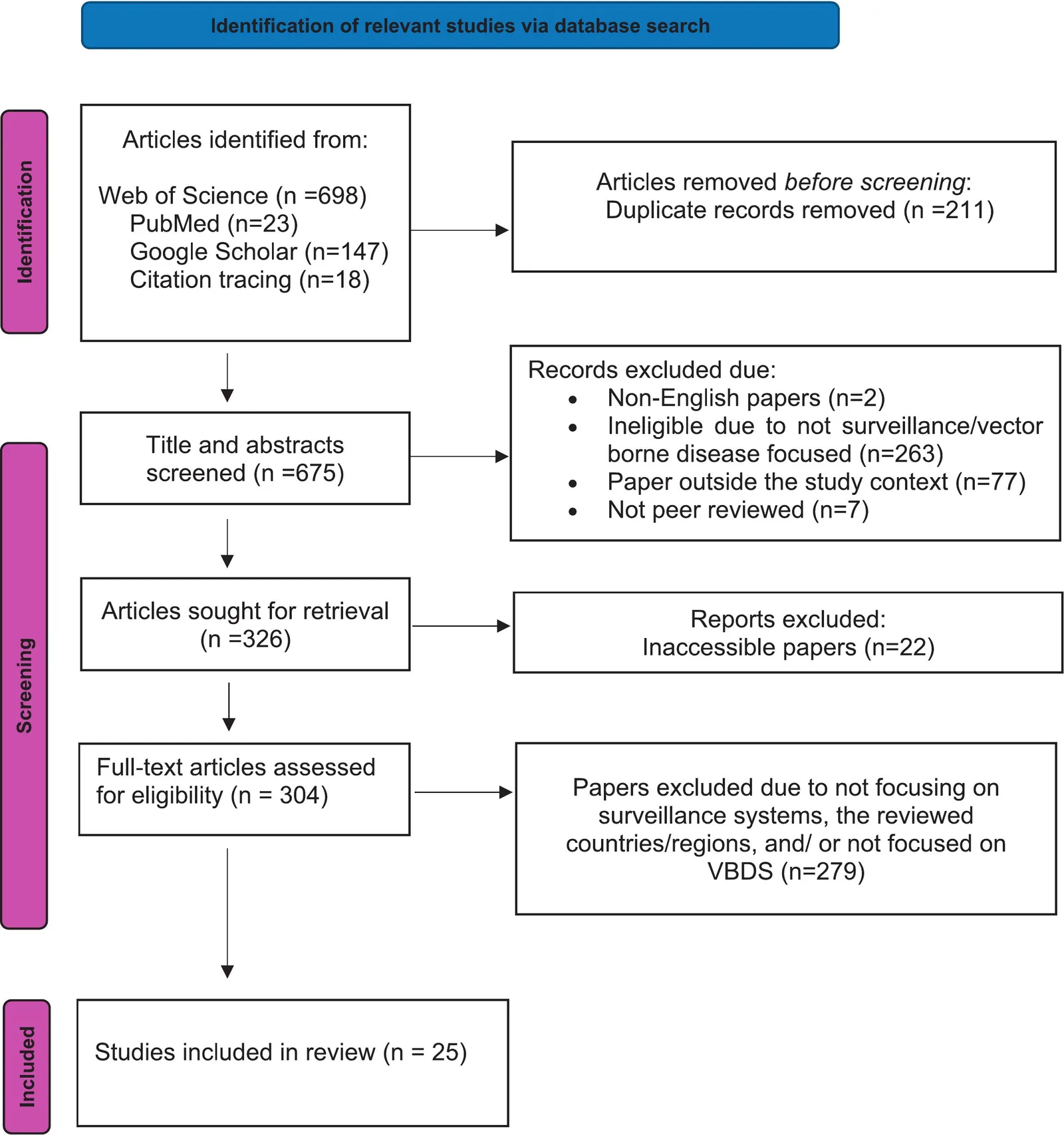
Flow diagram for the identification and selection protocol. Source: Adapted from the Preferred Reporting Items for Systematic Review and Meta-Analysis (PRISMA) protocol by Page et al. [43].

The evolving landscape of vector-borne disease surveillance in Europe reflects both the increasing recognition of environmental changes and the growing emphasis on integrated One Health approaches to disease prevention and control. In responding to this and following our systematic scoping searches and rigorous screening procedures, we identified a total of 25 studies (see Fig. 1 and Table S2 in Supplementary Material 2) that met our inclusion criteria for detailed analysis.

**Figs. 2–7:**
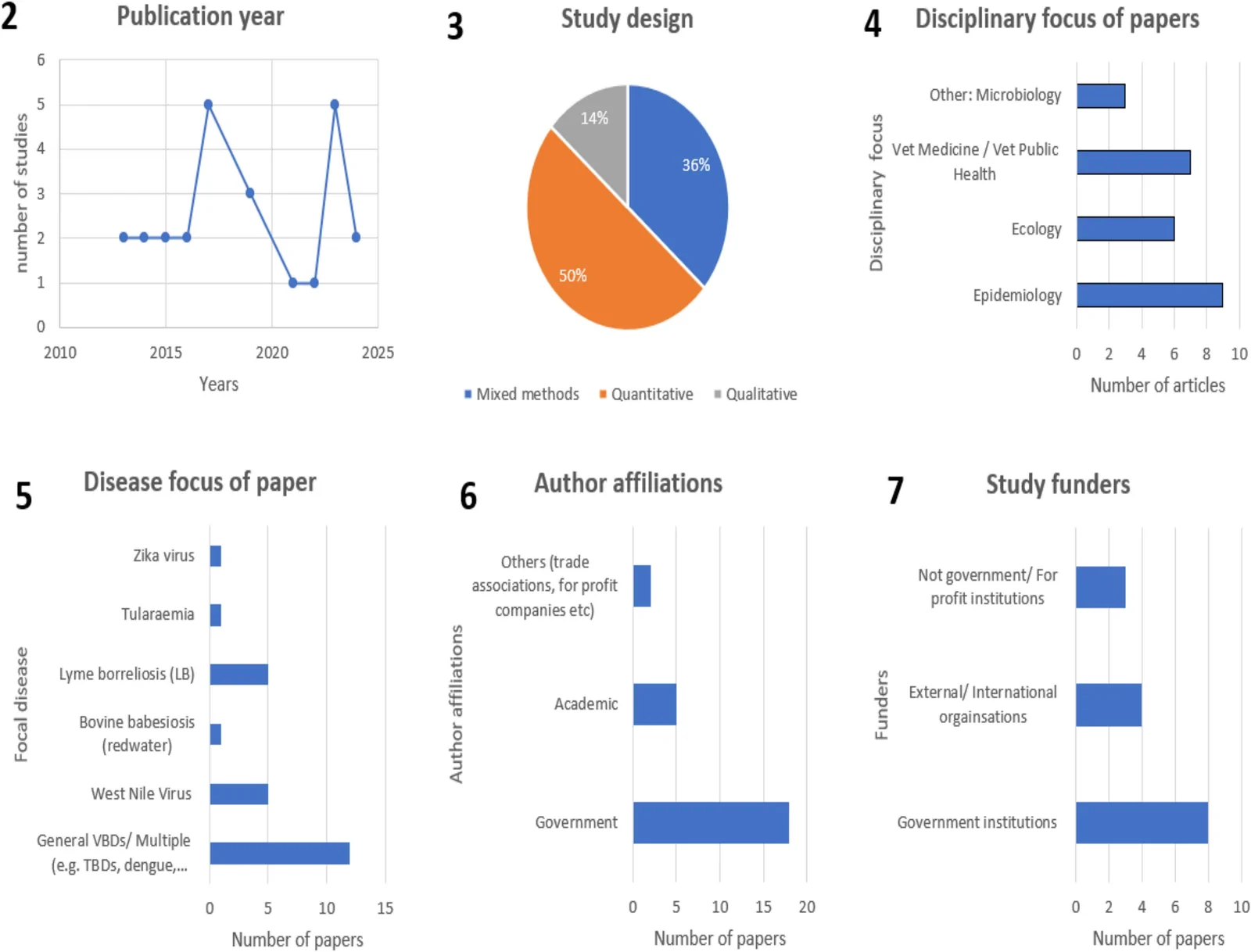
summary descriptives of the 25 reviewed articles. (2) for trends in publication over time (literature published between January 2012, and October 2025 were included). (3) Pie chart shows the number of publications by study design. Bar charts show the number of publications by (4) disciplinary focus, (5) focal disease, (6) author affiliation, and (7) study funder.

The majority of articles analysed (*n* = 17) were published between 2017 and 2024 (Fig. 2), suggesting an increasing interest in integrated surveillance approaches, perhaps as a response to broader trends toward the re-emergence of systems thinking in public health research observed over the past decade, rooted in a desire to redress the limitations of reductionist approaches to complex health challenges [45], [46]. This concentration can also be explained by or reflects the maturation of One Health concepts from theoretical frameworks to practical implementation approaches, coinciding with policy developments including the global One Health quadripartite initiative linked to infectious disease prevention (particularly pandemic preparedness), the European Commission's adoption of enhanced vector-borne disease preparedness measures and the implementation of the EU One Health Action Plan [47].

As shown in Fig. 3, the majority of publications were empirical studies (*n* = 21), of which just under half adopted predominantly quantitative approaches (*n* = 11). Notably, most (*n* = 10) of the quantitative studies conducted secondary data analysis of existing surveillance systems. This number increases to nearly three-quarters (18/25, 72%) for the overall corpus when other quantitative methods such as laboratory analysis, ecological sampling, surveys/questionnaires, or quantitative assessment frameworks are included. The strong quantitative focus can be explained by the nature of VBD surveillance research itself, which is fundamentally concerned with measuring and reporting disease incidence, prevalence, and geographic distribution patterns. Most studies sought to quantify risks and document pathogen circulation through (eco)epidemiological analyses, report outbreak incursions and case detections, describe monitoring activities of vectors and hosts, or model spatial-temporal disease patterns in vector and host populations. This also reflects the operational mandate of surveillance systems to generate measurable epidemiological data and insights that inform risk assessment and evidence-based public health interventions [48].

In disaggregating studies by their focal disease, most studies were not disease-specific, and instead examined multiple VBDs simultaneously such as Lyme disease, dengue, West Nile fever, Schmallenberg, and Bluetongue, or broader infectious disease surveillance incorporating non-vector-borne diseases such as bovine tuberculosis, avian influenza, and African swine fever (*n* = 12, 48%). A further four of these non-disease-specific studies focused specifically on monitoring tick and vector pathogen activities, which also cause vector-borne diseases. For those that were disease-specific, most papers focused on West Nile virus and Lyme borreliosis as the most frequently studied pathogens (*n* = 5, 20.8%) (see Fig. 5).

**Fig. 8:**
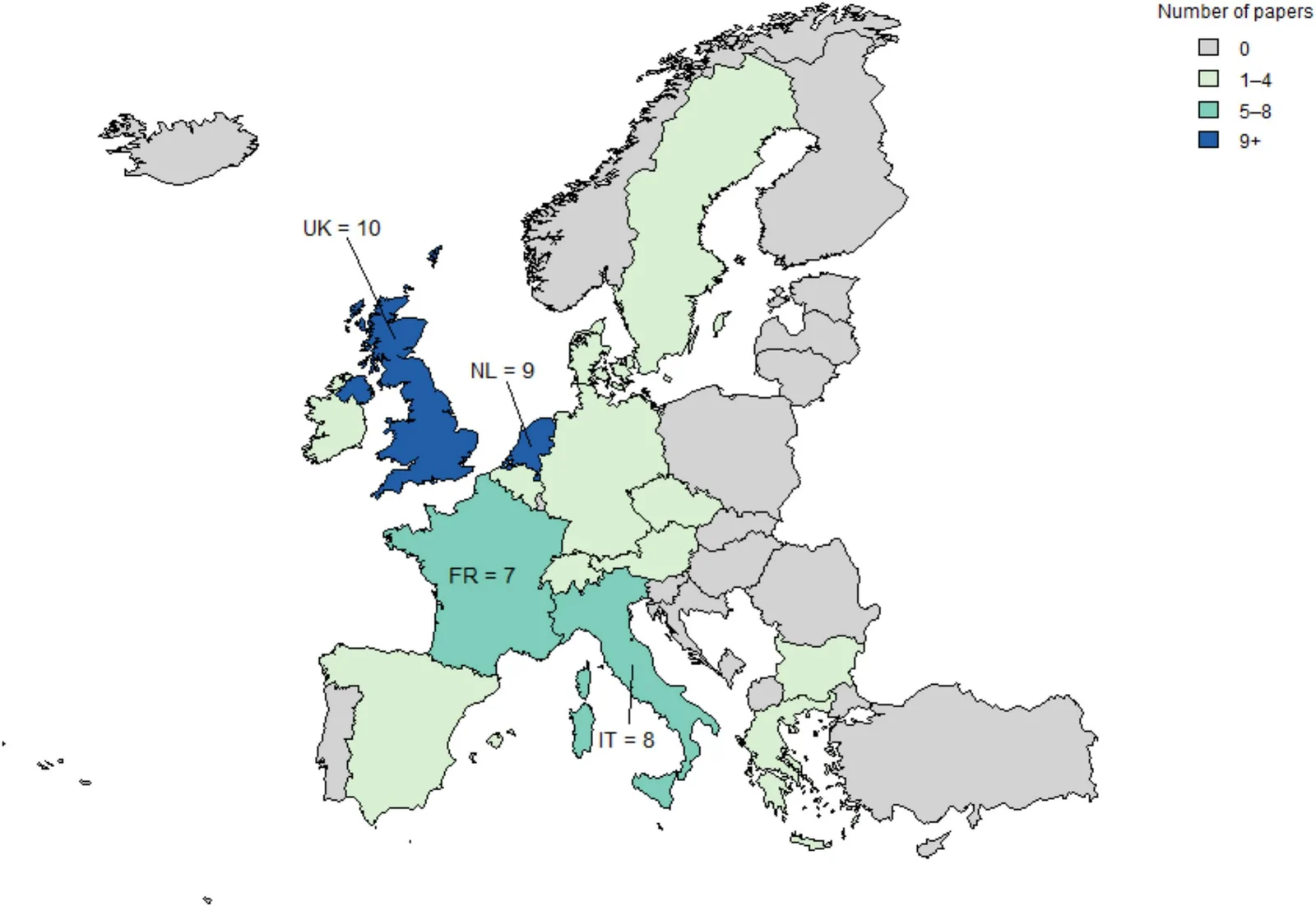
Focal locations for literature. Country counts represent individual extractions from reviewed studies. Countries appearing in multi-country studies are represented multiple times based on how many multi-country papers examined them.

In terms of geographical scope (see Fig. 8), majority of studies were either national or multi-national. The UK was the most frequently examined country, appearing in 10 studies (including 4 multi-country studies). This was followed by Italy, which was examined in 8 studies (including 4 multi-country studies and notably, 2 of these Italian studies were subnational in focus). France appeared in 7 studies (including 3 multi-country studies). An additional 7 papers examined multiple European countries, either through broad multi-country surveillance data or comparisons across a smaller number of named nations serving implicitly as primary case studies [39], [40], [49], [50], [51], [52], [53]. The predominance of UK-focused research may be partly explained by our focus on English-language publications. However, it may also reflect the country's substantial investment in surveillance infrastructure following major disease outbreaks, such as the 2001 foot and mouth crisis [54].

#### 3.1.2 Collaborations, one health approach, and transdisciplinarity in the reviewed studies

Analysis of the study designs reveals insights into the evaluation approaches used to assess surveillance systems. Unlike other areas such as infectious diseases and antimicrobial resistance (AMR) [e.g. [55], [56], One Health evaluation of VBD surveillance systems appears to be still nascent within the VBD surveillance literature. Of the 25 studies, only five [39], [49], [57], [58], [59], specifically utilised surveillance system evaluation frameworks and approaches, with four explicitly adapting One Health assessment evaluations. These included applications of evaluation protocol developed by the Network for Evaluation of One Health (NEOH) [49], [57] alongside other structured and process -evaluation approaches [39], [58], using interviews, surveys or questionnaires and focus groups/workshops to examine operationalisation, implementation and the effectiveness or One Health-ness^1^ of surveillance systems or initiatives. This limited attention to systematic evaluation suggests a potential gap between surveillance implementation and rigorous assessment of system performance.

Where identified, the analysis of academic disciplines shows a strong domination of epidemiology, public health and veterinary medicine (see Fig. 4). More than half of all papers (*n* = 16) were published by researchers from epidemiological and public health/veterinary medicine backgrounds. When technical practitioner authors are added, this increases to more than three-quarters of studies. Other disciplines represented included ecology and medical entomology. Notably absent were social science perspectives, which may explain the limited attention to socio-economic, political and stakeholder/community engagement dimensions of surveillance implementation.

As shown in Fig. 4, the disciplinary focus of published papers also reflected in publication outlet patterns. Though journal destinations for these articles were varied, with studies spread across 12 different publications in epidemiological, ecological and veterinary journals, this dispersion also points to fragmentation within the field. Studies appeared most frequently in the *International Journal of Environmental Research and Public Health (n = 4), Eurosurveillance (n = 3), Epidemiology & Infection (n = 3), Parasites & Vectors (n = 3), and Vector borne and Zoonotic diseases (n = 2)*, with single publications across the remaining journals. This dispersion suggests a lack of critical mass within any individual journal or discipline. Evidence of this fragmentation was found in limited cross-referencing between papers, indicating that researchers may be working in relative isolation within their respective disciplinary silos or surveillance systems. Whilst surveillance authors and specialists may naturally publish technical and epidemiological papers, the lack of a flagship journal^2^ or concentrated publication venue might also be suggestive of a limited cohesion necessary for effective knowledge synthesis and community building. Also, not only are social science and socio-ecological/socio-epidemiological perspectives in short supply, but there was also little evidence of interdisciplinary work that would bridge these disciplinary divides.

Of the 25 studies with funding disclosure information, 10 studies (40%) reported no funding, whilst 8 studies (32%) were funded by in-country government institutions such as DEFRA, the French Ministry of Agriculture, and the Welsh Government. Only 4 studies (16%) were funded by supra-national institutions like the European Union, with some limited private sector (Pfizer Inc., Bayer Animal Health) and professional body funding (*n* = 3, 12%). Similarly, focusing on institutional affiliations, there was a heavily government-dominated research landscape, with about three-quarters (72%, *n* = 18) of studies originating from government institutions. This overwhelming governmental presence includes national public health institutes, veterinary agencies, and disease control centres across Europe, with few contributions from academic institutions (*n* = 5, 20%) and private sectors (8%, *n* = 2) (also see Fig. 7).

This government dominance can be explained by the fact that surveillance systems originate and are mainly managed by key government agencies, hence driving related research (see section 4). Notably, this is also reflective of the prioritisation of specific vector-borne diseases and the need for data to underpin evidence-based public health interventions and disease control measures by governments. Some of these surveillance systems were commissioned as research projects by government agencies as an indicator-based measure of disease prevalence, incidence trends, or emerging pathogen circulation or in response to increased disease risks and emerging threats, with funding support earmarked by most country governments or subnational entities. From a surveillance and disease control perspective, governments have both the mandate and resources to invest in comprehensive monitoring systems that serve public health protection objectives. However, as noted in the disciplinary focus discussion, this heavy government concentration may reflect or explain the disciplinary silos observed earlier, though there was little overt exploration of this theme in the literature reviewed. Nevertheless, it opens up important opportunities for enhanced multi-sectoral collaboration, independent evaluation of surveillance system effectiveness, and strengthened One Health integration and research.

#### 3.1.3 Overview of surveillance characteristics and design in reviewed papers

Out of the papers reviewed, 20 studies explicitly focused on surveillance systems for vector-borne diseases. More than half addressed multiple VBDs, sometimes including broader infectious disease components or plans. Of these, 15 addressed human health surveillance exclusively, 12 examined animal health surveillance, and only 8 investigated environmental or ecological surveillance components. However, the depth of detail varied considerably. Just over half (*n* = 13) provided only generic descriptions such as ‘national surveillance plans’ without formal programme names or detailed operational information. These initiatives primarily operated within frameworks established at national, regional, or supranational levels, such as the French National Animal Health Surveillance Platform, multiple national West Nile virus programmes across countries, and European Centre for Disease Prevention and Control (ECDC) frameworks including VectorNet, the EVD-Net, and the EpEPIS platforms.

Only 7 studies provided sufficient detail about surveillance systems within documents reviewed to enable comprehensive characterisation of their One Health attributes and cross-sectoral design features, including evaluation or critical assessment of system performance. The following section therefore examines the VBD surveillance ecosystem to provide a broader picture of existing design, capacities and cross-sectoral integration.

### 3.2 Status and characteristics of VBD one health surveillance systems in Europe and the UK

Building on the literature review, this section presents our analysis of the broader VBD surveillance landscape. Through expanded desk-based analysis, we catalogued 72 distinct surveillance initiatives, which we characterise below in terms of operational design, policy objectives, disease prioritisation, and One Health integration.

#### 3.2.1 Surveillance characteristics and design

As shown in Table S3, analysis of the 72 European vector-borne disease surveillance systems revealed passive surveillance as the predominant approach, accounting for nearly half of all systems (*n* = 33) as exemplified by the UK's Tick Surveillance Scheme (https://www.gov.uk/guidance/tick-surveillance-scheme). Hybrid active-passive approaches were found in just over one in ten systems (*n* = 11), such as Italy's West Nile virus surveillance, which strategically deploy active surveillance in high-risk areas such as the Northern regions of Piedmont, Lombardy and Veneto, while maintaining passive reporting nationally. Roughly one in five systems operated solely through active surveillance, with these predominantly participatory initiatives such as Italy's Mosquitos Alert platform, the UK's Mosquito Recording Scheme and France's CiTIQUE citizen science platform for tick monitoring.

We identified surveillance systems or initiatives across 11 countries, including the UK, characterised by different spatial implementation patterns. National-level coordination was observed in over two-thirds of systems, demonstrating governmental recognition of vector-borne disease threats as national priorities requiring centralised public health responses. For instance, the UK and France operate through complementary national systems spanning human health, animal health and susceptible communities. Also, France's Sentinelles network leverages General Practitioner's (GP) reports, the French National Animal Health Surveillance Platform (NAHSP) provides comprehensive animal health surveillance, and CiTIQUE engages citizens in participatory tick monitoring. Each fills specific surveillance gaps while contributing to national disease intelligence. Only five systems operated at the subnational level only such as Italy's regional initiatives in West Nile virus and tick-borne disease surveillance. Multi-country regional systems comprised just under one-fifth of all systems (*n* = 11), predominantly ECDC-coordinated platforms such as VectorNet, EVD-Net, and EpiPulse, which facilitate European-level data sharing, early warning, sharing of standard operating protocols for surveillance activities, diagnostic harmonisation, and research coordination.

In terms of surveillance types, indicator-based surveillance (*n* = 30) was most common, followed by hazard-specific surveillance (*n* = 21) and general or syndromic surveillance approaches (*n* = 13). This pattern shows that data is often collected from defined human patient, livestock, wildlife, vector, or environmental sampling populations or sentinel sites to detect disease trends. Systems such as France's Sentinelles GP network and Spain's RENAVE epitomise indicator-based approaches, with the former tracking GP consultation patterns for febrile illness or specific syndromes, and the latter coordinating mandatory notification of confirmed vector-borne disease cases from clinicians and laboratories nationally. Conversely, hazard-specific surveillance, exemplified by the UK's Tick and Mosquito Recording Schemes, targets particular vectors or pathogens of concern, providing detailed ecological and distributional data but potentially missing emerging threats outside their defined scope.

While integrated surveillance platforms – in this context, systems embedded within broader institutional frameworks rather than operating independently – comprise the clear majority (*n* = 60) versus standalone systems (*n* = 12), most systems have evolved incrementally over the years, with periodic expansions and refinements responding to emerging threats, policy priorities, or integration within broader programmes, especially in the context of the UK [60]. Indeed, most standalone systems originated as research projects or targeted responses to specific threats (e.g. Mosquito Alert app in Spain, Tekenradar.nl in Netherland) designed to complement rather than replace official surveillance. Such trajectories from research-originated tools to components of national infrastructure illustrate the potential of the science-policy interface in translating innovations into operational practice. The broader landscape is nonetheless characterised by systems designed as integrated components within larger institutional frameworks. UKHSA for instance has progressively consolidated tick surveillance, mosquito monitoring, and invasive vector species detection within its surveillance programming [see [61]. Similarly, France's NAHSP evolved from disparate components into a unified platform from which additional system components and expertise, from which two more surveillance platforms were set up, one on plant health and the other on the food chain [70]. At the European level, ECDC's EpiPulse integrates previously separate systems, namely EPIS platforms for event-based epidemic intelligence, TESSy for indicator-based disease reporting across member states, and TTT for real-time outbreak tracking, into a comprehensive surveillance platform.

#### 3.2.2 Policy objectives and disease priorities

Across the identified systems (see Tables 2 and S3), multiple incentives rather than single motivating factors were noted as policy objectives. Analysis of stated objectives revealed public health protection as the primary institutional motivation, with a quarter of the systems combining both animal-public health objectives. Notably, only four systems explicitly prioritised wildlife health (APHA's DoWS, UK, France's SAGIR network, Portugal's ANIMAS and the Dutch Wildlife Health Centre), five prioritised animal or veterinary health exclusively — GD-Veekijker in the Netherlands, APHA's Tick-borne Disease Dashboard, Spain's RASVE, APHA's Bluetongue Surveillance Programme, and the French CIRAD OCAPI *Culicoides* monitoring network — and one prioritised environmental entomological monitoring (Rothamsted Insect Survey, UK), though animal, wildlife, and environmental components feature within many additional integrated systems. The limited number of systems with explicit non-human mandates likely stems from the predominant framing of surveillance as protecting human populations, with animal and wildlife surveillance justified primarily through their contribution to zoonotic disease prevention rather than as intrinsic health priorities.

**Table 2:** Overview characteristics of identified surveillance systems.

System name	Country	Spatial Scope	Surveillance type	Design	Ownership	Sectors Involved	Online Source / Reference
Belgium Sentinel Labs	Belgium	National	Indicator-based	Sentinel	Public	Human health	https://www.sciensano.be/en/projects/laboratory-data-networks-surveillance-infectious-diseases
Belgium's national One Health for Surveillance (OH4S) programme for WNV	Belgium	National	Early warning surveillance; Hazard-specific surveillance	Active	Public	Human health; Animal health; Wildlife	Sohier et al. [69]; Sohier et al. [71]
MosquitoSurveillance.be	Belgium	National	Participatory surveillance; Early warning surveillance	Active and passive surveillance	Public	Environment/Vector	sciensano.be/en/projects/monitoring-exotic-aedes-mosquitoes-belgium; mosquitosurveillance.be
Aktivita klistat Portal	Czech Republic	National	Hazard-specific	Active	Public	Human health; Environment	https://klistatavemeste.vsb.cz/en/
Sentinelles	France	National	Indicator-based; Syndromic	Sentinel	Public	Human health	https://www.sentiweb.fr/
SAGIR	France	National	General surveillance	Passive	Public	Wildlife	https://ofb.gouv.fr/sagir-le-reseau-de-surveillance-des-maladies-de-la-faune-sauvage
NAHSP (National Animal Health Surveillance Platform)	France	National	Early warning; Indicator-based; Risk-based	Active and Passive	Public	Animal health; Wildlife/ environmental; human health	https://www.plateforme-esa.fr/fr; Dupuy et al. [70]
CiTIQUE	France	National	Participatory	Active	Public	Human health; Animal health; Environment	https://www.citique.fr/
French National Culicoides Entomological Surveillance Programme / CIRAD OCAPI Database	France	National	Hazard-specific; Indicator-based	Active	Public	Animal health	http://ocapi.cirad.fr/
SurvStat@RKI	Germany	National	Indicator-based	Not applicable	Public	Human health	https://survstat.rki.de
SurvNet	Germany	National	Indicator-based	Passive	Public	Human health	Krause et al. [72]
National Reference Laboratory for CCHF and (separately NRL for WNV)- Friedrich-Loeffler-Institut	Germany	National	Hazard-specific surveillance		Public	Animal health; Human health (referral)	fli.de/en/institutes/institute-of-novel-and-emerging-infectious-diseases-innt/reference-laboratories/nrl-for-cchf/
WNV Integrated Surveillance	Italy	National	Early warning; Indicator-based; Hazard-specific	Active and Passive	Public	Human health; Animal health; Environment	Engler et al. [53]; Paternoster et al. [57]; Marchino et al. [58]; Rizzo et al. [73]
National TBD Surveillance System (Ministerial Decree 15/12/1990)	Italy	National	Indicator-based	Passive	Public	Human health	Garcia-Vozmediano et al. [49]
Regional TBE programmes	Italy	Subnational	Indicator-based	Passive	Public	Human health	Garcia-Vozmediano et al. [49]
ISS Network	Italy	National	Indicator-based	Passive	Public	Human health	Garcia-Vozmediano et al. [49]; https://www.iss.it/
IZS Network	Italy	National	Indicator-based; General surveillance	Passive	Private	Animal health	Garcia-Vozmediano et al. [49]; https://www.izs.it/IZS/Home_Page_en
EDSyS system for TBDs	Italy	Subnational	Hazard-specific; Syndromic	Active and Passive	Public	Human health; Animal health	Colucci et al. [74]
National integrated surveillance plan for some arboviral diseases	Italy	National	Early warning; Indicator-based; Hazard-specific	Active and Passive	Public	Human health; Animal health; Environment	https://www.epicentro.iss.it/en/arboviral-diseases/surveillance
Regional WNV Surveillance Programs (Piedmont, Veneto, Emilia-Romagna, others)	Italy	Subnational	Hazard-based; Indicator-based	Active	Public	Human health; Animal health; Environment	Marchino et al. [58]; Gobbo et al. [75]
Dutch Animal Health Surveillance System (AHSS)	Netherlands	National	General surveillance	Active and passive	Public	Animal health	https://www.gdanimalhealth.com/en/Disease-control/MonitoringSurveillance; Santman-Berends et al. [76]
GD-Veekijker	Netherlands	National	General surveillance	Passive	Public	Animal health	https://www.gdanimalhealth.com/en/Disease-control/MonitoringSurveillance; Santman-Berends et al. [76]
RIVM surveillance systems (including TBD)	Netherlands	National	Indicator-based; General surveillance	Not applicable	Public	Human health	Garcia-Vozmediano et al. [49]; Maas et al. [77]; https://www.ecdc.europa.eu/en/rijksinstituut-voor-volksgezondheid-en-milieu-rivm; https://www.rivm.nl/en/infectious-disease-control/strengthening-infectious-disease-surveillance
Tekenradar.nl	Netherlands	National	Indicator-based; Participatory	Passive	Public	Human health	https://www.tekenradar.nl/
Tekenscanner app	Netherlands	National	Hazard-specific; Participatory	Passive	Private	Animal health	Jongejan et al. [78]
RIVM-CIb programme	Netherlands	National	Indicator-based; General surveillance	Not applicable	Public	Human health	Maas et al. [77]; https://www.rivm.nl/en/international-projects/surveillance4nl
Dutch Signalling Forum Zoonoses	Netherlands	National	Early warning; Risk-based	Not applicable	Public	Human health; Animal health	https://www.rivm.nl/en/one-health/dutch-signalling-forum-zoonoses
REVIVE - Rede de Vigilancia de Vetores	Portugal	National	General surveillance; Hazard-specific surveillance; Point-of-entry surveillance (IHR)	Active	Public	Environment/Vector	https://www.insa.min-saude.pt/revive-rede-de-vigilancia-de-vetores-relatorio-2024/#
ANIMAS (Aplicacao de Notificacao Imediata de Mortalidade de Animais Selvagens)	Portugal	National	Participatory surveillance; Event-based surveillance	Passive	Public	Wildlife	https://www.dgav.pt/animais/conteudo/animais-selvagens/notificacao-de-animais-mortos/
SIVIZ (Sistema Integrado de Vigilancia e Alerta para Zoonoses)	Portugal	National	Early warning surveillance; Hazard-specific surveillance	Active	Public	Animal health; Wildlife and human health	https://www.dgav.pt/informacaoutil/content/programas-e-projetos/siviz/; https://health.ec.europa.eu/funding/eu4health-programme-2021-2027-vision-healthier-european-union_en
ECDC VectorNet	Europe	Regional (multi-country)	Hazard-specific	Active and Passive	Public	Human health; Animal health	https://www.ecdc.europa.eu/en/about-us/partnerships-and-networks/disease-and-laboratory-networks/vector-net
ECDC EVD-Net	Europe	Regional (multi-country)	Early warning; Risk-based	Not applicable	Public	Human health	https://www.ecdc.europa.eu/en/about-ecdc/partners-and-networks/disease-and-laboratory-networks/european-emerging-and-vector-borne
ECDC EPIS (in EpiPulse)	Europe	Multi- Regional (multi-country)	Event-based	Not applicable	Public	Human health	https://www.ecdc.europa.eu/en/publications-data/epipulse-european-surveillance-portal-infectious-diseases
ECDC TTT (in EpiPulse)	Europe	Regional (multi-country)	Event-based; Risk-based	Not applicable	Public	Human health	https://www.ecdc.europa.eu/en/publications-data/epipulse-european-surveillance-portal-infectious-diseases
ECDC TESSy (in EpiPulse)	Europe	Regional (multi-country)	Indicator-based	Not applicable	Public	Human health	https://www.ecdc.europa.eu/en/publications-data/epipulse-european-surveillance-portal-infectious-diseases
EpiPulse	Europe	Regional (multi-country)	Indicator-based; Event-based	Not applicable	Public	Human health	https://www.ecdc.europa.eu/en/publications-data/epipulse-european-surveillance-portal-infectious-diseases
ECDC E3 Hub	Europe	Regional (multi-country)	Risk-based	Not applicable	Public	Human health; Environment	https://geoportal.ecdc.europa.eu/e3-network/generaldescription/
ECDC Geoportal	Europe	Regional (multi-country)	Indicator-based	Not applicable	Public	Human health	https://geoportal.ecdc.europa.eu/e3-network/generaldescription/
EU-IBIS	Europe	Regional (multi-country)	Indicator-based	Passive	Public	Human health	https://www.ecdc.europa.eu/en/about-us/networks/disease-and-laboratory-networks/eu-ibd
Epitweetr	Europe	Regional (multi-country)	Syndromic; Event-based	Passive	Public	Human health	https://www.ecdc.europa.eu/en/publications-data/epitweetr-tool
OH SURVector (One Health surveillance and Vector monitoring for cross-border pathogens)	Europe	Regional (multi-country)	Early warning surveillance; Hazard-specific surveillance	Active	Public	Environment/Vector	ages.at/ohsurvector; EU4Health Grant 101,132,974
Romania TBE system	Romania	National	Indicator-based	Passive	Public	Human health	https://insp.gov.ro/
National Epidemiological Surveillance Network (RENAVE)	Spain	National	Indicator-based	Passive	Public	Human health	https://www.healthinformationportal.eu/health-information-sources/national-epidemiological-surveillance-network
CCAES	Spain	National	Early warning; Event-based	Not applicable	Public	Human health	https://www.sanidad.gob.es/areas/alertasEmergenciasSanitarias/reglamentoSI/cne.htm
Andalusian WNV Surveillance system	Spain	Subnational	Hazard-based	Active and Passive	Public	Human health; Animal health; Environment	https://salutpublica.gencat.cat/ca/ambits/vigilancia/mdo-a-z/febre-del-nil-occidental-vno/index.html#googtrans(ca|en)
Mosquito Alert App and portal	Spain	National	Hazard-based; Participatory	Active	Public	Human health; Environment	https://www.mosquitoalert.com/en/; https://map.mosquitoalert.com/en-US/reports?mosquitoes=albopictus,culex&from = 2014-01-01&to = 2026-07-30
Galician Network for Surveillance of Vector-Borne Diseases (ReGaViVec)	Spain	Subnational	Hazard-based	Active, Sentinel	Public	Human health; Animal health; Environment	https://www.mosquitoalert.com/en/press-release-the-xunta-confirms-the-presence-of-tiger-mosquitoes-in-the-municipality-of-bueu-through-the-galician-network-for-surveillance-of-vector-borne-diseases-regavivec/
Catalonia WNV surveillance (humans, domestic animals, wild birds)	Spain (Catalonia)	Subnational	Early warning surveillance; Hazard-specific surveillance	Active and passive surveillance	Public	Human health; Animal health; Wildlife	salutpublica.gencat.cat; Busquets et al. [79]
RASVE - Red de Alerta Sanitaria Veterinaria	Spain	National	Event-based surveillance; Indicator-based surveillance	Passive	Public	Animal health	https://www.mapa.gob.es/en/ganaderia/temas/sanidad-animal-higiene-ganadera/sanidad-animal/alertas-sanitarias/redes_vigilancia https://www.mapa.gob.es/en/ganaderia/temas/sanidad-animal-higiene-ganadera/sanidad-animal/
Sentinella	Switzerland	National	Indicator-based; Syndromic	Sentinel	Public	Human health	https://www.idd.bag.admin.ch/en/survey-systems/sentinella
Tick Prevention app of Switzerland	Switzerland	National	Participatory	Passive	Public	Human health	https://zecke-tique-tick.ch/en/
Tick Surveillance Scheme	UK	National	Hazard-specific; Enhanced passive; Participatory	Passive	Public	Human health; Animal health	https://www.gov.uk/guidance/tick-surveillance-scheme
Mosquito Recording Scheme	UK	National	Hazard-specific; Participatory	Passive	Public	Human health; Animal health	https://www.gov.uk/guidance/mosquitoes-how-to-report; Vaux and Medlock. [63]
Nationwide Mosquito Survey	UK	National	Hazard-specific	Active	Public	Human health	https://www.gov.uk/government/publications/mosquito-surveillance/mosquito-nationwide-surveillance; Vaux and Medlock. [63]
APHA's Scanning Surveillance Network	UK	National	General surveillance	Passive	Public	Animal health	https://www.gov.uk/guidance/animal-disease-scanning-surveillance-at-apha
SAVSNET	UK	National	Syndromic	Passive	Private	Animal health	https://www.liverpool.ac.uk/savsnet/
APHA VIDA database	UK	National	General surveillance	Passive	Public	Animal health	https://www.gov.uk/guidance/view-apha-surveillance-reports-publications-and-data
UKHSA Dashboard	UK	National	General surveillance	Not applicable	Public	Human health	https://ukhsa-dashboard.data.gov.uk/
Finger Tips	UK	National	General surveillance	Not applicable	Public	Human health	https://fingertips.phe.org.uk
Vet Compass	UK	National	General surveillance	Passive	Private	Animal health	https://www.rvc.ac.uk/vetcompass
APHA DoWS	UK	National	General surveillance	Passive	Public	Wildlife	https://www.gov.uk/guidance/animal-disease-scanning-surveillance-at-apha
UKHSA Real-time Syndromic	UK	National	Syndromic	Passive	Public	Human health	https://ukhsa-dashboard.data.gov.uk/syndromic-surveillance
Remote health advice syndromic	UK	National	Syndromic	Passive	Public	Human health	https://www.gov.uk/government/publications/remote-health-advice-weekly-bulletins-for-2026
UKHSA RIPL	UK	National	Indicator-based	Passive	Public	Human health	https://www.gov.uk/government/collections/rare-and-imported-pathogens-laboratory-ripl
EDSyS England	UK	National	Syndromic	Passive	Public	Human health	https://www.gov.uk/government/collections/syndromic-surveillance-systems-and-analyses
BPSU/UKOSS	UK	National	Indicator-based	Sentinel	Public	Human health	https://www.npeu.ox.ac.uk/ukoss
APHA Tick-borne disease dashboard	UK	National	Indicator-based	Not stated	Public	Animal health; Environment	https://public.tableau.com/app/profile/siu.apha/viz/Tick-bornediseasestory/TBD
UK Invasive mosquito surveillance scheme	UK	National	Hazard-specific	Active	Public	Human health; Environment	https://www.gov.uk/guidance/invasive-mosquito-surveillance;
UK National Tick Pathogen Survey	UK	National	Hazard-specific	Active	Public	Human health	https://www.gov.uk/guidance/national-tick-survey; Vaux and Medlock. [63]
Rothamsted Insect Survey	UK	National	Hazard-specific	Not stated	Public	Environment; Animal health	https://www.rothamsted.ac.uk/national-capability/the-insect-survey
APHA Bluetongue Surveillance Programme	UK	National	Indicator-based	Not stated	Public	Animal health	https://www.gov.uk/government/news/bluetongue-disease-control-framework-set-out
VB-RADAR (Vector-Borne Real-time Arbovirus Detection And Response)	UK	National	Early warning surveillance; Hazard-specific surveillance	Active	Public	Animal health; Wildlife; Human health (via UKHSA)	Bruce et al. [80]; UKHSA et al. [82]

Surveillance objectives clustered around four primary functions: just over half of the systems aimed at early detection for rapid response (*n* = 42) or trend monitoring for intervention design and evaluation (*n* = 32), and just under one-third (*n* = 22) for detection of emerging pathogens. The latter was particularly evident in systems with robust laboratory components such as RIVM in the Netherlands and UKHSA's Rare and Imported Pathogens Laboratory, which maintain capacity for novel pathogen identification. Notably, fewer systems (*n* = 7) explicitly aimed at early detection for risk prediction. While analytical methods are emerging, this gap in predictive surveillance capabilities may be partly explained by the multi-focus nature of most systems, given that predictive models typically target specific diseases with well-characterised transmission dynamics such as TBE and Lyme disease, potentially limiting the feasibility of disease-specific forecasting.

As expected, given their fundamental role in transmission, vector surveillance featured in nearly two-thirds of the systems, whether through active monitoring, passive recording, or integration within multi-component systems. For instance, the UK's Mosquito Recording Scheme and Nationwide Mosquito Survey exemplify dedicated vector surveillance, monitoring for invasive *Aedes* species that could establish dengue or chikungunya transmission if climatic conditions become favourable [62], [63]. However, the majority of these vector surveillance systems captured seasonal and spatial variability in vector abundance rather than variability in vector infection rates or contact rates with human or animal populations. Human pathogen surveillance (*n* = 31), awareness activities, and vector pathogen surveillance (*n* = 22) were also noted across systems, with programmes such as Germany's mosquito monitoring testing specimens for West Nile virus, *Usutu* virus, and other arboviruses to detect viral circulation before human cases emerge. Implementation of wildlife host surveillance (*n* = 13), livestock host surveillance for human VBDs (*n* = 6) and environmental pathogen monitoring (*n* = 7) remained limited though this partially stems from the historically reductionist approach to disease control focussing solely on human treatments without addressing environmental reservoirs of pathogens [64]. While systems such as France's SAGIR network and the Dutch Wildlife Health Centre demonstrate the value of wildlife surveillance for detecting disease emergence before human spillover, such systems remain in the minority relative to human-focused approaches.

Relatedly, though assessment was complicated by the multi-disease focus nature of many systems — as noted above, for most systems (*n* = 48, 65%) that were general or multi-focus rather or oriented toward broad vector and pathogen activity monitoring, we identified tick-borne diseases (particularly Lyme borreliosis and tick-borne encephalitis) and mosquito-borne diseases (notably West Nile virus) as the most frequently prioritised across systems (see disease matrix in Table S5 in Supplementary Material 3).

Among disease-specific systems, tick-borne diseases, notably Lyme borreliosis and TBE, were the most explicitly targeted. Consistent with EFSA priority rankings, Lyme borreliosis had dedicated systems in the UK and the Netherlands, reflecting its status as the most prevalent vector-borne disease in Europe [65], [66]). *Borrelia burgdorferi* sensu lato was the most frequently targeted pathogen across disease-specific systems (*n* = 5), though many additional multi-focus systems include Lyme surveillance within broader tick-borne disease monitoring. TBE represented another key priority, predominantly in endemic regions, including Italy's Regional TBE Surveillance and systems in the Czech Republic and Romania, and with case counts increasing into previously non-endemic areas such as the UK [16]. Notably, however, Germany which reports among the highest TBE case burdens in western Europe lacked any dedicated TBE-specific system in the identified literature, suggesting a potential documentation or retrieval gap, or reliance on statutory notification pathways not captured by our search.

Others, such as West Nile virus, featured in approximately one in six systems (*n* = 12) with a specific WNV focus, including Italy's integrated WNV surveillance network and components of Italy's National Arbovirus Plan, though WNV monitoring also occurs within many multi-focus arbovirus surveillance systems, particularly in Mediterranean countries that have experienced increasing outbreaks and where environmental and ecological conditions favour transmission [67], [68]. Indeed, West Nile virus represented the only pathogen with dedicated emerging disease surveillance, primarily concentrated in countries such as Italy and Spain, where significant WNV outbreaks have occurred and where climatic and ecological conditions favour established vector-competent *Culex* mosquito populations. Notably, few systems such as Italy's National Arbovirus Plan and Belgium's West Nile surveillance, explicitly integrated endemic and emerging disease surveillance within a single framework.

The veterinary VBD landscape though limited showed a complementary but largely separate pattern, with bluetongue and Schmallenberg surveillance concentrated in the UK, Netherlands and France through dedicated vector entomological monitoring programmes, notably the APHA Annual Bluetongue Surveillance Programme, the Netherlands GD-Veekijker, and the French CIRAD OCAPI *Culicoides* monitoring network. Others, including Tick-borne Fever (*Anaplasma phagocytophilum*), louping ill, and tick pyaemia and redwater, received explicit dedicated coverage only within the UK veterinary surveillance framework, despite their recognised burden in livestock-rearing regions across the British Isles and Scandinavia. Conversely, other exotic mosquito-borne threat surveillance noted as key in some systems (e.g. dengue, chikungunya, Zika), particularly in the UK and Spain, may reflect a precautionary or biosecurity logic given that these systems are primarily aimed at early detection of invasive vector establishment and imported case amplification.

#### 3.2.3 Dimensions and degrees of one health-ness of VBD surveillance systems in Europe and UK

To assess One Health-ness – integration and regulatory coherence across Human Health, Public Health and Environment sectors – we examined how governance arrangements facilitate or constrain coordinated, multi-sectoral approaches across three levels: (i) policy-level integration (ii) institutional-level coordination and (iii)operational-level collaboration (see Tables S6 to S8 in Supplementary Material 3).

At the policy level, despite over 90% public funding and national government leadership in system operations, sectoral integration remained limited. Indeed, about three-quarters of systems operated under single-sector mandates. These were predominantly human health led by public health agencies like UKHSA, RIVM, Santé Publique France or animal health, led by veterinary authorities like UK's APHA. About ten of the systems had dual-sector mandates (UK Tick Surveillance Scheme, Dutch Signalling Forum for Zoonoses), while explicit human-animal-environmental mandates characterised only by about 7 systems. Environmental health sector inclusion remained limited (*n* = 14), representing a major governance gap given environmental determinants fundamentally shape transmission dynamics of VBDs. In practice, this could include incorporating habitat and land-cover data, or climate-linked risk modelling, or environmental agency involvement in coordination structures, as seen in a small number of the systems we sourced. The predominance of human health mandates perhaps stems from historical surveillance organisation within public health ministries, with animal and environmental sectors engaged primarily when zoonotic transmission necessitates cross-sectoral coordination. National-level frameworks were also the most common (*n* = 58), with subnational mandates rare and multi-country frameworks comprising (*n* = 11, ECDC platforms), though European implementation varied across member states due to different legal frameworks and institutional structures as well as potentially different disease priorities [23], [66].

Translating policy mandates into operational reality required institutional coordination mechanisms, which characterised more than two-thirds of systems (*n* = 55), though depth and formality varied considerably. Research institutes and veterinary services were involved in less than half of systems, providing specialised expertise (e.g. INRAE in CiTIQUE, CIRAD in NAHSP, university partnerships in SAVSNET) or private or public veterinary services. Cross-sectoral coordination bodies were found to enable integration in exemplary cases such as Italy's West Nile virus surveillance operating through Ministry of Health oversight with ISS coordination, integrating regional health authorities, veterinary institutes (IZS network), and environmental monitoring. Others such as the Frances NAHSP incorporates 11 institutions across sectors with specialised working groups including the Epidemic Intelligence group combining animal and human health expertise; the UK's HAIRS group brings together UKHSA, APHA, and environmental agencies for zoonotic risk assessment, though functioning more as advisory mechanism than for operational coordination. We also found likely documentation of formal coordination mechanisms – inter-agency agreements, memoranda of understanding, or legislative mandates – in a subset of systems, with ECDC providing European-level coordination through technical advisory groups and data governance structures, yet institutional implementation remained heterogeneous across member states.

At the operational level, where day-to-day surveillance activities occur, data exchange (including data collation) mechanisms were most common (across most systems), though nature ranged from unidirectional reporting to effective bidirectional information flow. Inter-sectoral data analysis occurred in just over a quarter of the 72 reviewed systems exemplified by Italy's West Nile virus surveillance combining human case data, veterinary reports, wild bird surveillance, and entomological monitoring to generate integrated risk assessments informing public health interventions and blood safety measures. Where identified, laboratory resource sharing and/or diagnostic protocol harmonisation occurred at similar frequency (*n* = 20) while joint protocol development (*n* = 10) and coordinated sampling campaigns (*n* = 12) remained limited. The predominance of data exchange may be explained by the relative ease of information sharing compared to harmonising operational procedures across institutions with different mandates, interest, capacities, and organisational cultures. For instance, while ECDC's technical standards and data harmonisation protocols facilitate European-level operational coordination and protocol sharing, the practical implementation across countries depends on national institutional arrangements and resource availability, underscoring that governance arrangements, not technical capacity alone, fundamentally shape One Health integration potential.

## 4 Conclusions

In this review, we present the first evidence synthesis and mapping of the One Health VBD surveillance systems landscape across Europe and the UK. Our synthesis revealed several overarching patterns that provide ready opportunities for a future research agenda.

While the concentration of recent publications, particularly post-2017, signals growing recognition of integrated surveillance approaches, this temporal clustering may reflect increased policy attention following high-profile outbreaks across Europe – West Nile virus expansion, tick-borne disease emergence – rather than fundamental shifts in surveillance philosophy or practice alone [66]. The maturation of One Health concepts from theoretical frameworks to practical implementation, coinciding with European policy developments such as the EU One Health Action Plan, has undoubtedly catalysed research interest. Whilst many studies investigated drivers of disease emergence, burden, and transmission rather than simply describing surveillance systems, the limited application of formal evaluation frameworks nonetheless suggests that rigorous independent assessment of surveillance system performance, effectiveness, and One Health integration remains underrepresented relative to operational and descriptive work. This observation underscores an urgent need for future research that moves beyond epidemiological or social science investigation of disease systems and impacts from surveillance and surveillance system descriptions toward critical evaluation of system performance, effectiveness, and One Health integration using standardised assessment tools such as the NEOH evaluation protocol or adapted WHO frameworks, or more socio-economic assessments or evaluations of surveillance effectiveness and operationalisation [[20], [81], [83]]. Longitudinal studies tracking surveillance system evolution and measuring outcomes of integration efforts would provide essential evidence for understanding which One Health approaches yield measurable improvements in disease detection, response capacity, or resource efficiency across different institutional and political contexts.

The geographical concentration of research efforts – with the UK appearing in a substantial proportion of studies or systems – raises important questions about generalisability and representation in the surveillance evidence base. While our focus on English-language publications partially explains this predominance, this geographical skewness/bias risks creating an evidence landscape that privileges well-resourced Northern or Northwestern European contexts while underrepresenting surveillance approaches, challenges, and innovations from Southern, Central, and Eastern European countries where vector-borne disease burdens may be highest and resource constraints most acute. For instance, the relative absence of published evaluations from Mediterranean and Eastern European countries – regions experiencing active West Nile virus circulation and tick-borne encephalitis expansion – though may be shaped by our search strategy, represents a key knowledge gap. Future research efforts should prioritise partnerships with surveillance institutions across diverse European contexts, particularly those in endemic regions or countries with emerging vector-borne disease threats, to ensure the evidence base reflects the full spectrum of surveillance approaches, capacities, and constraints operating across the continent. Comparative multi-country studies examining how different governance structures, resource levels, and institutional arrangements shape One Health implementation would yield invaluable insights for strengthening surveillance systems region-wide.

In analysing the literature profile, we also noted the relative lack of and hence opportunity for more social science research, including sociopolitical, biocultural and interdisciplinary sociotechnical perspectives. The dominance of epidemiological, public health, and veterinary perspectives reflects the technical nature of surveillance work but also highlights key gaps in understanding the socio-political dimensions of cross-sectoral collaboration, community engagement in surveillance design, and community priorities for surveillance and interventions. This is not to undermine the importance of quantitative driven epidemiological research in surveillance design, development and implementation; however, it does suggest that such work could be substantially enhanced through deeper engagement with social science approaches. Indeed, the disciplinary fragmentation extends beyond methodological preferences to fundamental epistemological differences about how health threats should be conceptualised and addressed for different societal outcomes. If surveillance systems are to meaningfully embrace One Health principles – which by definition demand integration across human, animal, and environmental health domains – the research community studying these systems must itself become more transdisciplinary. Mixed methods approach combining quantitative-inspired epidemiological analysis with qualitative investigation of institutional cultures, stakeholder perspectives including community vulnerability and adaptive capacity, and implementation barriers would provide in-depth understanding of factors enabling or constraining One Health integration beyond technical interoperability.

As we have shown, perhaps most revealing is the heavy government dominance in authorship and ownership, with the majority of studies originating from government institutions. While this pattern reflects the reality that surveillance systems originate from and are managed by government agencies as we noted earlier, it raises questions about whether independent, critical evaluation of surveillance system performance — distinct from the quality assessment that peer review provides — is sufficiently resourced and prioritised. Government-led research may prioritise system description and operational reporting, perhaps over critical analysis of structural limitations, resource inefficiencies, or governance failures. The concentration of funding from in-country government institutions versus supranational bodies like the European Union suggests research remains nationally focused despite the transboundary nature of vector-borne disease threats. While this apparent fragmentation potentially hinders development of harmonised European approaches and limits opportunities for cross-national learning, it also presents opportunities for future research. The field would benefit from cross-institutional and inter-sectoral funding involving independent academic institutions, with capacity for collaboratively designed and independently conducted evaluation of surveillance system effectiveness, cost-efficiency, and One Health integration, working alongside government agencies and international bodies. This is already evident where research-originated projects have been embedded within national surveillance infrastructure, and an indicator of an effective science-policy interface. Realising this potential more broadly will require improved science-policy interfaces and dedicated funding for shared understanding of surveillance questions and needs.

Also, the operational characteristics of identified surveillance systems reveal a seeming persistent tension between ‘integrated’ One Health aspirations and sectoral implementation realities. Despite widespread rhetoric emphasising cross-sectoral collaboration, this enthusiasm has not translated into adoption of effective cross-sectoral surveillance frameworks, as seen in the majority single-sectoral focus of studies and systems and the limited depth of cross-sectoral collaboration beyond data collection and sharing. While such focus may reflect resource constraints and institutional path dependencies that favour established, single-sector surveillance over more resource-intensive integrated approaches, it presents key gaps, particularly in the level of effective One Health-ness and integration with environmental sectors. Also, though we acknowledge the significant difficulty of resolving fundamental conceptual confusion surrounding One Health terminology, future research can make meaningful progress through pragmatic approaches. Rather than seeking universal definitional consensus, we suggest two next steps. First, comparative studies across multiple systems could classify collaboration by type and depth, distinguishing for example data exchange alone from joint sampling or joint analysis, rather than relying on a single binary “integrated or not integrated” label. Second, evaluation frameworks should be tailored to a system's own context, its sectors, scale and governance structure, assessing not only whether collaboration occurred but its quality and its effect on decisions and health outcomes.

Following the contributions of this review, we also acknowledge some limitations which themselves present areas for further research. First, despite our multi-disciplinary team, our focus on English-language publications and on One Health- or integration-specific search terms likely contributed to UK overrepresentation and may have excluded relevant research published or systems in other European languages, or pathogen-specific studies, particularly from Southern and Eastern European countries. Future studies should address this linguistic and terminology bias through multilingual, pathogen-specific search strategies and partnerships with non-Anglophone research institutions. Second, whilst our inclusion criteria for the literature synthesis required peer-reviewed publications as a quality control mechanism, our grey literature and institutional searches, though broader, were also not exhaustive. Indeed, VBD surveillance systems likely exist across Europe – including marked heterogeneities in arrangements between UK devolved administrations – but remain undocumented in the sources we were able to access. Due to time and access constraints, we could not undertake systematic appraisal of grey literature, particularly governmental agency databases which require negotiated access [44]. Future studies should incorporate systematic grey literature reviews and primary data collection from national surveillance institutions, along with stakeholder expert interviews, to capture the full landscape of surveillance practice. Third and relatedly, our ability to comprehensively characterise One Health attributes was constrained by limited detail provided in many papers, with only a minority providing sufficient information for comprehensive characterisation. This necessitated supplementary desk-based research to catalogue the full landscape of 72 surveillance systems. This approach, while enabling broader coverage, may have introduced inconsistencies in characterisation depth across systems, hence calling for more primary data collection and deeper engagement with surveillance practitioners to understand operational realities.

Overall, this review adds to our theoretical and practical understanding of the potential and limits of One Health implementation in surveillance systems, providing an evidence base to guide more intentional, rigorously evaluated approaches to integrated vector-borne disease surveillance across diverse European contexts. As environmental changes drive the increasing emergence and burdens of zoonotic and vector-borne diseases, closing the gap between One Health aspirations and operational implementation becomes not merely academically interesting but epidemiologically urgent. The seemingly disconnect between One Health rhetoric and operational realities documented in this review raises an open question for future research, about what governance and institutional changes would be needed for more effectively integrated surveillance capable of responding to complex, transboundary vector-borne disease challenges.

## CRediT authorship contribution statement

**Godfred Amankwaa:** Writing – review & editing, Writing – original draft, Software, Methodology, Investigation, Formal analysis, Data curation, Conceptualization. **Emmanuel S. Tomude:** Writing – review & editing, Methodology, Data curation. **Hermann Kam:** Writing – review & editing, Data curation. **Bethan V. Purse:** Writing – review & editing, Project administration, Funding acquisition, Conceptualization. **Jonathan Rushton:** Project administration, Funding acquisition, Conceptualization. **Mara Rocchi:** Project administration, Funding acquisition, Conceptualization. **Kayleigh Hansford:** Writing – review & editing, Project administration, Funding acquisition, Conceptualization. **Jolyon M. Medlock:** Writing – review & editing, Funding acquisition. **Nicholas Johnson:** Writing – review & editing, Funding acquisition, Conceptualization. **Festus A. Asaaga:** Writing – review & editing, Writing – original draft, Project administration, Methodology, Investigation, Funding acquisition, Data curation, Conceptualization.

## Funding

This research in this paper has been supported by the environmental solutions to reduce the risk of current and future tick-borne zoonotic pathogens in the UK (TickSolve) project funded by 10.13039/100014013UK Research and Innovation (UKRI) through the 10.13039/501100000270Natural Environment Research Council (NERC) (grant numbers NE/W003260/1, NE/W003171/1, NE/W003244/1 and NE/W003252/1), and the OPTICK project by the 10.13039/501100000268Biotechnology and Biological Sciences Research Council (BBSRC), NERC, UKRI Tackling Infections Strategic Theme and Department for Environment Food & Rural Affairs (Defra) (grant no. BB/X017974/1).

## Declaration of competing interest

The authors declare no competing interest.

## Data Availability

Data has been uploaded

## Footnotes

1 By one health-ness, we refer to the degree of cross-sectoral integration and regulatory coherence across policy (sectoral mandates and governance), institutional (coordination mechanisms and participation), and operational (data exchange, joint analysis, resource sharing, protocol harmonisation, and coordinated sampling) levels of surveillance systems

2 Eurosurveillance may see itself as this journal but the disciplinary range is limited
