# Supplementary material for: Mapping one health surveillance and networks for vector-borne disease prevention and control in Europe – A scoping review
Source: One Health. 2026 Aug 18;23:101545. doi: 10.1016/j.onehlt.2026.101545 (PMC13545381; doi:10.1016/j.onehlt.2026.101545)
Supplement: Supplementary file 1 [file mmc4.pdf]

New OH Scoring framework - Integrated OneHealth surveillance systems for vector-borne diseases in the UK

SYSTEMATIC SCOPING REVIEW: INTEGRATED "ONE HEALTH" SURVEILLANCE SYSTEM IN THE UNITED KINGDOM - CHARACTERISING AND MAPPING THE SURVEILLANCE ECOSYSTEM AND COLLABORATIVE NETWORKS FOR PREVENTION AND CONTROL OF VECTOR-BORNE DISEASES THIS SYSTEMATIC SCOPING REVIEW SEEKS TO CHARACTERISE AND MAP THE EXISTING SURVEILLANCE AND CONTROL SYSTEMS, NETWORKS AND POLICY FRAMEWORKS FOR VECTOR-BORNE DISEASE MANAGEMENT IN THE UK, WITH THE VIEW OF IDENTIFYING INTEGRATION POINTS, GAPS AND OPPORTUNITIES FOR INTEGRATED ONE HEALTH SURVEILLANCE SYSTEM. TO OUR KNOWLEDGE, THERE IS NO RECENT REVIEW OF THE STAKEHOLDER ECOSYSTEM AND SURVEILLANCE NETWORKS AND PROGRAMMES FOR VECTOR-BORNE DISEASES IN THE UK. USING A MIXED METHODS APPROACH COMPRISING A DOCUMENT REVIEW OF PEER-REVIEWED AND GREY LITERATURE, KEY INFORMANT INTERVIEWS AND SURVEY QUESTIONNAIRES WITH SELECTED CROSS-SECTORAL STAKEHOLDERS ACROSS THE UK, WE AIM TO: IDENTIFY CURRENT VBD SURVEILLANCE INITIATIVES, ACTORS, INTERACTIONS AND ROLES IN SURVEILLANCE AND MONITORING SYSTEMS, EXPLORE STAKEHOLDER PERCEPTIONS ABOUT STRENGTHS, WEAKNESSES, OPPORTUNITIES AND THREATS FOR EXISTING SURVEILLANCE SYSTEM CHARACTERISE PRIORITY VECTOR-BORNE DISEASES FOR CROSS-SECTORAL STAKEHOLDERS IN THE UK, AND ASSESS NATIONAL AND EU CONTROL STRATEGIES AND RISK ASSESSMENTS FOR INDIVIDUAL DISEASES/SUITES OF DISEASE.

INITIAL QUESTIONS GUIDING THE REVIEW HAVE BEEN DEVELOPED AND WILL BE REFINED BASED ON RESEARCH TEAM DISCUSSIONS AND FEEDBACK RECEIVED FROM CROSS-SECTORAL STAKEHOLDERS. WHAT NATIONAL AND REGIONAL VECTOR- AND TICK- BORNE DISEASE SURVEILLANCE ECOSYSTEMS, NETWORKS, PROGRAMMES EXIST OR ARE PLANNED? WHO ARE THE RELEVANT CROSS-SECTORAL STAKEHOLDERS, AND ACTORS AND WHAT ARE THEIR PRIORITIES FOR AND POTENTIAL ROLES IN AN INTEGRATED "ONE HEALTH" SURVEILLANCE SYSTEM? WHICH STAKEHOLDERS WERE ENGAGED IN THE DEVELOPMENT OF EXISTING SURVEILLANCE AND CONTROL SYSTEMS, AND HOW WERE THEY ENGAGED? WHAT DOMAINS AND TYPES OF DATA ARE COLLECTED? HOW DECISIONS ARE MADE ABOUT COLLATION, USE AND INTEGRATION INTO INFORMATION SYSTEMS AND WHAT IS THE DECISION-MAKING PROCESS AMONG WHICH KEY ACTORS? WHAT ARE THE MECHANISMS FOR DATA FLOW AND GOVERNANCE OF DISEASE CONTROL MEASURES? WHAT ARE THE CURRENT PERCEIVED DATA OR EVIDENCE GAPS OR BIASES AFFECTING DECISION MAKING? HOW DO DIFFERENT STAKEHOLDERS DEFINE THE "SUCCESS" OF INTEGRATED ONE HEALTH SURVEILLANCE SYSTEMS? HOW DO SURVEILLANCE AND CONTROL SYSTEMS FOR EXOTIC AND ENDEMIC VECTOR-BORNE INFECTIONS INTERACT AND ARE THERE OPPORTUNITIES TO LEVERAGE DATA, KNOWLEDGE, RESOURCES AND INFRASTRUCTURE BETWEEN THESE SYSTEMS?

1. REVIEWER DETAILS:

2. DATE OF ENTRY:

3. DOCUMENT TYPE:

- ◯ 1= Empirical article
- ◯ 2= Review article
- ◯ 3= Policy document
- ◯ 4=Other (please specify)

3.1 IF OTHER, PLEASE SPECIFY:

4. ADD URL OF DOCUMENT:

....

5. JOURNAL/ ORGANISATION NAME WHERE PAPER IS PUBLISHED:

....

6. YEAR OF PUBLICATION OF THE ARTICLE/DOCUMENT:

....

7. PLEASE SPECIFY THE STUDY DESIGN:

- ◯ 0= Not mentioned
- ◯ 1= Qualitative
- ◯ 2= Quantitative
- ◯ 3= Mixed methods
- ◯ 4= others

7.1 IF OTHERS, SPECIFY

....

8. WHAT IS THE RESEARCH METHOD(S) USED?

- ☐ 0=Not mentioned
- ☐ 1= Interviews
- ☐ 2= Focus Group Discussion
- ☐ 3= Workshops
- ☐ 4= Review
- ☐ 5= Other (please specify)
- ☐ 99= Not applicable

8.1 IF OTHER, PLEASE SPECIFY:

....

SURVEILLANCE SYSTEM OR DISEASE CONTROL PROGRAMME/INITIATIVE

9.1. EXPLICITNESS OF SURVEILLANCE SYSTEM/ INITIATIVE FROM PAPER

Note if the surveillance system is clearly defined or just mentioned or suggested but undefined

- ◯ 1= Explicit (Clearly defined)
- ◯ 2= Implicit (Suggested or mentioned but undefined)

9.2 NAME OF THE INITIATIVE/ PROGRAMMES:

Note: Please enter all surveillance/ disease programmes listed in the document

....

10. TYPE OF INITIATIVE/PROGRAMME REPORTED:

- [ ] 1= Surveillance system
- [ ] 2= Disease control system
- [ ] 3= Vector control programme
- [ ] 4= Other intervention system

11. WHICH ASPECTS OF DISEASE MANAGEMENT ARE CAPTURED IN THE SURVEILLANCE OR DISEASE CONTROL PROGRAMME?

Tick as many as apply

- [ ] 1= Human treatment
- [ ] 2= Treatment of livestock
- [ ] 3= Human vaccination
- [ ] 4= Vaccination of livestock as reservoirs
- [ ] 5= Vaccination of wildlife
- [ ] 6= Awareness raising among health professionals
- [ ] 7= Awareness raising among the public
- [ ] 8= Vector surveillance
- [ ] 9= Vector control for population reduction
- [ ] 10= Vector eradication
- [ ] 11= Disease eradication
- [ ] 12= Pathogen surveillance in humans
- [ ] 13= Pathogen surveillance in livestock
- [ ] 14= Pathogen surveillance in wildlife
- [ ] 15= Pathogen surveillance in vectors
- [ ] 16= Pathogen surveillance in the environment
- [ ] 17= Modelling of disease risk
- [ ] 18= Modelling of intervention strategies

12. PURPOSE/OBJECTIVE(S) OF SURVEILLANCE PROGRAMME/INITIATIVE:

Select as many as apply

- ☐ 1= early detection for rapid response
- ☐ 2= trends monitoring to support intervention design/evaluation
- ☐ 3= early detection for rapid risk prediction
- ☐ 4= trends monitoring for eradication/control
- ☐ 5= early detection for timely response
- ☐ 6= early detection for rapid risk assessment
- ☐ 7= trends monitoring to improve knowledge
- ☐ 8= detect new pathogen/diseases or unusual epidemiological events
- ☐ 9= estimate the magnitude of a health problem
- ☐ 10= evaluate and/or improve official surveillance systems
- ☐ 11= evaluate control or eradication strategies
- ☐ 12= demonstrate freedom from a particular pathogen/infection
- ☐ 13= Other (please specify)

12.1 IF OTHER, PLEASE SPECIFY:

....

12.2 PURPOSE OF VECTOR CONTROL OR MANAGEMENT:

- ☐ 1= Eradicate the vector
- ☐ 2= Reduce the population extent of the vector
- ☐ 3= Reduce the biting rate or abundance of the vector in human habitation
- ☐ 4= Reduce the biting rate or abundance of the vector in livestock habitation / farms

13. PURPOSE OF DISEASE CONTROL INITIATIVE:

- [ ] 1= Awareness raising
- [ ] 2= Reduce human exposure
- [ ] 3= Reduce human disease effects
- [ ] 4= Reduce animal exposure (as susceptible)
- [ ] 5= Reduce animal disease effects
- [ ] 6= Reduce vector populations
- [ ] 7= Reduce vector biting
- [ ] 8= Reduce vector breeding habitat
- [ ] 9= Reduce host populations
- [ ] 10= Reduce host-human interactions
- [ ] 11= Reduce host-animal interactions
- [ ] 12= Reduce host infection rates
- [ ] 13= Reduce host spread
- [ ] 14= Reduce vector spread
- [ ] 15= Other (please specify)

13.1 IF OTHER, PLEASE SPECIFY:

....

14. INCENTIVES FOR DISEASE SURVEILLANCE/CONTROL:

- [ ] 1= Protect animal health
- [ ] 2= Protect public health
- [ ] 3= Protect animal and public (human) health
- [ ] 4= Safeguard food security
- [ ] 5= Support rural economy
- [ ] 6= Compliance with international obligations & requirements
- [ ] 7= Trade considerations
- [ ] 8= not applicable/ mentioned

14.1 PLEASE ADD FURTHER INFORMATION ON THE INCENTIVES FOR DISEASE SURVEILLANCE/CONTROL:

....

15. YEAR OF ESTABLISHMENT/IMPLEMENTATION OF INITIATIVE/PROGRAMME:

....

15.1 REFERENCE OR WEBSITE (IF AVAILABLE):

....

16. WAS THE SURVEILLANCE OR CONTROL PROGRAMME INITIATED IN RESPONSE TO PARTICULAR OUTBREAK EVENTS?

- 0=No
- 1=Yes

16.1 IF SO, WHICH DISEASE IN WHICH HOST AND OUTBREAK YEARS? WHY IN TERMS OF CHANGE IN PERCEIVED THREATS?

17. TYPE OF INTERFACE (E.G. OPEN-ACCESS DASHBOARD/PROPRIETARY SOURCE):

- 1= Open-access dashboard
- 2= Proprietary source
- 3= both

18. OPERATIONAL STATUS (E.G. OPERATIONAL, IN-DEVELOPMENT, DORMANT):

- 1= Operational
- 2= In-development
- 3= Dormant/inactive
- 4= Others/ not applicable

18.1. IF OTHER, SPECIFY

19. AGENCY(IES) INITIATING OR RUNNING THE PROGRAMME/INITIATIVE:

Please list all the agencies involved in the establishment/ running of the surveillance/ disease control programme/initiative

19.1 PLEASE IDENTIFY THE ROLES OF THE IDENTIFIED AGENCIES/ INSTITUTIONS IN RESPECT OF SURVEILLANCE AND/OR DISEASE CONTROL:

20. STATUS OF THE SURVEILLANCE/CONTROL SYSTEM:

- 1= Stand-alone (autonomous)
- 2= Part of a bigger surveillance/control programme/initiative

20.1 NAME OF LINKED PROGRAMME/INITIATIVE:

If part of a larger programme/initiative, please state:

21. GEOGRAPHICAL COVERAGE (TERRITORY UNDER SURVEILLANCE):

22. PLEASE STATE THE INTENSITY AND HABITAT/HOST/PREMISE FOCUS OF THE SURVEILLANCE:

23. NUMBER OF SITES SURVEILLED (IF INFORMATION IS AVAILABLE):

....

24. IS THE SAMPLING SEASONAL SNAPSHOT OR MONTHLY?

- ○ 1= Seasonal snapshot
- ○ 2= Monthly
- ○ 3 = other

25. IS THE SAMPLING SEASONAL SNAPSHOT OR MONTHLY:

....

26. GEOGRAPHICAL GRANULARITY:

- ☐ 1= Subnational
- ☐ 2= National
- ☐ 3=Supra-national
- ☐ 4=Regional
- ☐ 5=Multi-scale

27. SECTORAL AFFILIATION (E.G. HUMAN HEALTH, ANIMAL HEALTH, WILDLIFE)

- ☐ 1=Human health
- ☐ 2= Animal health
- ☐ 3= Wildlife
- ☐ 4= Environment
- ☐ 5= Animal/human
- ☐ 6= Human/Animal/Environment
- ☐ 7= Human/Environment
- ☐ 8= Animal/Environment
- ☐ 9= Other
- ☐ 99= Not applicable

27.1. IF OTHER, PLEASE SPECIFY

....

Design of surveillance or control programme

28. SURVEILLANCE DESIGN:

Select as many as apply - e.g. surveillance could be both active and have sentinels

- ☐ 1= Active
- ☐ 2= Passive
- ☐ 3= Sentinel
- ☐ 4= Active and passive surveillance
- ☐ 5= Other
- ☐ 99= Not applicable

28.1. IF OTHER, SPECIFY

29. SURVEILLANCE TYPES:

- ☐ 1= Early warning surveillance
- ☐ 2= Indicator-based surveillance
- ☐ 3= Hazard-specific surveillance
- ☐ 4= General surveillance
- ☐ 5= Syndromic surveillance
- ☐ 6= Event-based surveillance
- ☐ 7= Risk-based surveillance
- ☐ 8= Enhanced passive surveillance
- ☐ 9= Participatory surveillance
- ☐ 10= Other
- ☐ 99= Not mentioned/ not applicable

29.1. IF OTHER, PLEASE SPECIFY

30. SAMPLING STRATEGY (INCLUDING SPATIAL, HOST OR HABITAT TARGETING IF RELEVANT AND LEVELS OF SPATIAL AND SEASONAL REPLICATION):

30.1 SAMPLING DESIGN (E.G. RISK-BASED, RANDOM, STRATIFIED SAMPLING):

- ○ 1= Risk-based sampling
- ○ 2= Random sampling
- ○ 3= Stratified sampling
- ○ 4= Other

30.1.1 IF OTHER, SPECIFY

31. SPATIAL EXTENT:

32. SURVEILLANCE COVERAGE:

Select as many as apply

- ☐ 1= Pathogen surveillance in humans
- ☐ 2= Pathogen surveillance in livestock
- ☐ 3= Pathogen surveillance in wildlife
- ☐ 4= Pathogen surveillance in vectors
- ☐ 5= Pathogen surveillance in the environment
- ☐ 6= Vector surveillance on hosts
- ☐ 7= Vector surveillance in the environment
- ☐ 8= Others
- ☐ 99= Not mentioned/ not applicable

33. TYPE OF PREMISES IN WHICH SURVEILLANCE OR CONTROL IS TARGETED:

Select as many as apply

- ☐ 1= Farm
- ☐ 2= Housing/ Residential settings
- ☐ Hospitals
- ☐ 3= Nature reserve
- ☐ 4= Other (please specify)

33.1 IF OTHER PREMISES, PLEASE SPECIFY:

34. BROAD HABITATS OR LAND COVERS INCLUDED IN THE SURVEILLANCE OR CONTROL PROGRAMME (IF KNOWN):

CONTENT

35. FOCAL PATHOGEN/AGENT OF INTEREST:

36. FOCAL SUSCEPTIBLE HOSTS INCLUDED IN THE SURVEILLANCE OR CONTROL PROGRAMME:

37. FOCAL TRANSMISSION HOSTS INCLUDED IN THE SURVEILLANCE OR CONTROL PROGRAMME:

E.g. livestock, pets, wild mammals non-game, wild mammals & game, wild birds, humans

38. FOCAL DISEASES THE SURVEILLANCE OR CONTROL PROGRAMME COVERS:

....

39. VECTORS INCLUDED IN THE SURVEILLANCE OR CONTROL PROGRAMME:

....

40. VECTOR HABITATS INCLUDED IN THE SURVEILLANCE OR CONTROL PROGRAMME:

....

41. STATUS OF THE DISEASE IN THE AREA OF INTEREST (E.G. ENDEMIC, EPIDEMIC, EXOTIC-NOT YET ARRIVED)

- ○ 1= Endemic
- ○ 2= Epidemic
- ○ 3= Exotic (not yet arrived)
- ○ 4= other

IF OTHER, SPECIFY

....

42. HOW MANY DISEASES DOES THE SURVEILLANCE/CONTROL PROGRAMME COVER?

....

43. TYPE OF DISEASE HAZARDS (E.G. VECTOR-BORNE, FOOD-BORNE, WATER-BORNE)

- ☐ 1= Vector-borne
- ☐ 2= Food-borne
- ☐ 3= Water-borne

44. NUMBER OF DOMAINS(I.E. COMPARTMENTS OF TRANSMISSION) UNDER SURVEILLANCE:

....

45. TYPE OF DATA COLLECTED IN EACH DOMAIN:

....

46. DATA SOURCE IN EACH DOMAIN (E.G. HOSPITAL DATA, VETERINARY PRACTICE DATA, COMMUNITY DATA)

- ☐ 1= Hospital data
- ☐ 2= Veterinary practice data
- ☐ 3= Community data
- ☐ 4= Not specified
- ☐ 5= Others

46.1. IF OTHERS, PLEASE SPECIFY

....

47. TYPES OF POPULATIONS INCLUDED (E.G. HEALTHCARE FACILITIES, VET PRACTICES, FARMS, COMMUNITY)

- [ ] 1= Healthcare facilities
- [ ] 2= Vet practices
- [ ] 3=Farms
- [ ] 4= Human dwellings
- [ ] 5= Schools
- [ ] 6= Environmental sources (e.g. waste-water)
- [ ] 7=Community
- [ ] 8= Other populations (please specify)

47.1 IF OTHER POPULATIONS, PLEASE SPECIFY:

REGULATORY ENVIRONMENT

48. DISEASE NOTIFICATION STATUS:

- ○ 1=Notifiable
- ○ 2=Non-notifiable
- ○ 99= Not mentioned

49. IF NOTIFIABLE, SPECIFY IN THE CONTEXT OF HUMAN OR ANIMAL HEALTH:

- ○ 1= Human health
- ○ 2=Animal health
- ○ 3=Both

50. IS THE SURVEILLANCE/ CONTROL INITIATIVE WRITTEN INTO LEGISLATION/POLICY?

- ○ 0=No
- ○ 1=Yes
- ○ 99= Not mentioned

50.1 IF YES, PLEASE SPECIFY LEGISLATION/POLICY:

51. IS THE FUNDING STREAM FOR SURVEILLANCE/ CONTROL PROGRAMME BACKED BY LEGISLATION/ POLICY?

- ○ 0=No
- ○ 1=Yes
- ○ 99=Not mentioned

GOVERNANCE/COORDINATION OF THE SURVEILLANCE SYSTEM/DATA

52. OWNERSHIP TYPE (E.G. PUBLIC, PRIVATE, PUBLIC-PRIVATE):

- ◯ 1= Public
- ◯ 2= Private
- ◯ 3= Public-private

53. GOVERNANCE MECHANISM OF INITIATIVE/PROGRAMME (NUMBER OF INSTITUTIONS IN CHARGE OF THE COORDINATION):

....

54. TYPES OF INSTITUTIONS/ACTORS INVOLVED IN THE COORDINATION/COLLABORATION (E.G. GOVERNMENT, ACADEMIA, INDEPENDENT AGENCY, ETC.)

Select as many as apply

- ☐ 1= Government
- ☐ 2= Research institutes
- ☐ 3= Independent agency
- ☐ 4= Official laboratories
- ☐ 5= Public health services
- ☐ 6= Hospitals/doctors
- ☐ 7= Private veterinarians
- ☐ 8= Universities
- ☐ 9= Agricultural sector
- ☐ 10= Environmental agencies
- ☐ 11= Wildlife management
- ☐ 12= Wildlife rescue centres
- ☐ 13= Pharmaceutical companies
- ☐ 14= Hunting sector
- ☐ 15= Other

54.1. IF OTHER, SPECIFY

....

55. HOW GOVERNANCE MECHANISM VARIES BY TYPE OF END USER (E.G. NATIONAL GOVERNMENT, LOCAL GOVERNMENT, ACADEMIC PARTNER, INDUSTRY):

- [ ] 1= National government
- [ ] 2= Local government
- [ ] 3= Academic partner
- [ ] 4= Industry
- [ ] 5= Public
- [ ] 6= Other (please specify)

55.1 IF OTHER, PLEASE SPECIFY:

....

56. FREQUENCY OF DATA COLLECTION (E.G. CONTINUOUS, MONTHLY, QUARTERLY, ANNUALLY):

- ○ 1= Continuous
- ○ 2=Monthly
- ○ 3= Quarterly
- ○ 4= Annually
- ○ 5= Seasonal snapshot
- ○ 6= Other

56.1 IF SEASONAL SNAPSHOT, PLEASE SPECIFY THE SEASONS FOR DATA COLLECTION:

....

56.2. IF OTHER, SPECIFY

....

57. SECTOR/S INVOLVED (E.G. HUMAN HEALTH, ANIMAL HEALTH, AGRICULTURE, WILDLIFE)

- [ ] 1= Human health
- [ ] 2= Animal health
- [ ] 3= Agriculture
- [ ] 4= Wildlife
- [ ] 5= Other (please specify)

57.1 IF OTHER, PLEASE SPECIFY:

....

58. TYPES OF DATA USERS (E.G. VETS, FARMERS)

....

58.1 WHICH AGENCIES AND END-USERS HAVE ACCESS TO THE SURVEILLANCE/ DISEASE CONTROL INFORMATION AND FOR WHICH PURPOSES (GIVEN THEIR ROLE IN THE HEATH OR ENVIRONMENT SYSTEM)?

E.g. Research institutes, public health services, hospitals, private veterinarians, official laboratories etc.

59. GROUPS/TEAMS RESPONSIBLE FOR PASSIVE DATA COLLECTION (ON TICKS, LIV, AP, BABESIA):

60. GROUPS RESPONSIBLE FOR SURVEILLANCE DATA MANAGEMENT (E.G. FOR MANAGING THE COLLATION OF DATA):

61. GROUPS RESPONSIBLE FOR OTHER ASPECTS OF INTERVENTIONS (SUCH AS VECTOR CONTROL, AWARENESS RAISING ETC.):

62. GROUPS RESPONSIBLE FOR SURVEILLANCE DATA INTERPRETATION:

62.1 FOR WHOM AND WHAT PURPOSE IS THE DATA BEING INTERPRETED?

Could also add anticipated uses of surveillance data by different user groups

63. ARE RESEARCH ORGANISATIONS INVOLVED IN THE SURVEILLANCE OR DATA INTERPRETATION?

- ◯ 0= No
- ◯ 1= Yes
- ◯ 99= Not mentioned/ not known

63.1 IF YES, PLEASE SPECIFY:

64. TO WHOM ARE THE RESULTS DISSEMINATED?

65. FREQUENCY OF DISSEMINATION OF THE RESULTS (E.G. MONTHLY, QUARTERLY, ANNUALLY):

- ◯ 1= Daily
- ◯ 2= Weekly
- ◯ 3= Fortnightly
- ◯ 4= Monthly
- ◯ 5= Quarterly
- ◯ 6= Annually
- ◯ 99= Not mentioned/ not known

66. CONTRIBUTION TO A SUPRANATIONAL MONITORING PROGRAMME:

- ◯ 0= No
- ◯ 1= Yes
- ◯ 99= Not mentioned/ not known

66.1 IF YES, PLEASE SPECIFY THE SUPRANATIONAL MONITORING PROGRAMME:

....

FUNDING AND SUSTAINABILITY

67. ORIGIN OF FUNDS FOR THE SURVEILLANCE PROGRAMME/INITIATIVE (STATE, PRIVATE, EXTERNAL, ETC.):

- ☐ 1= National government
- ☐ 2= European
- ☐ 3= Regional
- ☐ 4= Private
- ☐ 5=National and European
- ☐ 6= National and International
- ☐ 7= National public and private
- ☐ 8= No fund
- ☐ 9= Not applicable

68. SUSTAINABILITY OF FUNDING (I.E. DURATION IN YEARS, CYCLE LENGTH):

- ○ One time study
- ○ 1 year
- ○ 2 years
- ○ 2.5 years
- ○ 3 years
- ○ 4 years
- ○ 5 years
- ○ 6 years
- ○ 7 years
- ○ 8 years
- ○ 10 years
- ○ 14 years
- ○ 15 years
- ○ 20 years
- ○ Every second year
- ○ Every 3 years
- ○ Every 5 years
- ○ Continuous

COLLABORATION TYPOLOGY

69. DECISION-MAKING SCALES INVOLVED IN SURVEILLANCE ACTIVITIES (SUPRA-NATIONAL AUTHORITIES/ORGANISATIONS, NATIONAL AUTHORITIES, SUB-NATIONAL AUTHORITIES, ETC.)

- [ ] 1= Supra-national authorities/organisations
- [ ] 2= National authorities
- [ ] 3= Sub-national authorities
- [ ] 4= Other (please specify)

69.1 SPECIFY THE LEVEL SUB-NATIONAL DECISION-MAKING (E.G. LOCAL HEALTH AUTHORITIES):

69.2 IF OTHER, PLEASE SPECIFY:

70. TYPES OF COLLABORATIVE EFFORTS FOR SURVEILLANCE/CONTROL ACTIVITIES:

- [ ] 1= Conception of the surveillance protocol
- [ ] 2= Joint sampling campaigns
- [ ] 3= Laboratory facilities sharing
- [ ] 4= Data exchange
- [ ] 5= Inter-sectoral data analysis and interpretation
- [ ] 6= Sharing diagnostic protocols
- [ ] 7= Other (please specify)

70.1 IF OTHER, PLEASE SPECIFY:

71. MECHANISMS IN PLACE TO SUPPORT COLLABORATION FOR SURVEILLANCE ACTIVITIES:

72. TYPES OF COLLABORATIVE EFFORTS FOR INTERPRETATION AND DISSEMINATION OF SURVEILLANCE RESULTS:

73. MECHANISMS IN PLACE TO SUPPORT COLLABORATION FOR DISSEMINATION OF SURVEILLANCE RESULTS:

74. TYPES OF DISCIPLINES INVOLVED IN THE SURVEILLANCE PROCESS:

75. FACILITATORS OF COLLABORATION:

E.g. initiatives, facilities, interests, legislation, personnel in charge

76. BARRIERS TO COLLABORATION:

E.g. initiatives, facilities, interests, legislation, personnel in charge

77. PERCEIVED STRENGTHS OF THE SURVEILLANCE OR CONTROL SYSTEMS:

78. PERCEIVED WEAKNESS OF THE SURVEILLANCE OR CONTROL SYSTEMS:

79. IS/ARE RISK OR PREDICTIVE MODELS USED AS PART OF THE SURVEILLANCE OR CONTROL PROGRAMME?

- 0= No
- 1= Yes

79.1 IF SO, FOR WHAT PURPOSE ARE THE MODELS DEVELOPED?

79.2 WHAT ARE THE KEY MODEL OUTPUTS LINKED TO THIS PURPOSE?

79.3 DO THE MODELS DRAW DIRECTLY ON DATA FROM THE SURVEILLANCE SYSTEM?

- 0= No
- 1= Yes

79.4 DO THE MODELS INCLUDE THE FOLLOWING COMPARTMENTS IN THEIR FORMULATION?

- 1= Vector
- 2= Livestock
- 3= Wildlife
- 4= Human
- 5= Environment

79.5 IS THERE EVIDENCE OF DIRECT USE OF MODEL OUTPUTS BY DECISION MAKERS TO DATE?

- 0= No
- 1= Yes

79.6 IF SO, WHAT WAS THE IMPACT ON DECISION MAKING?

EVALUATION OF ONE HEALTH THINKING IN SURVEILLANCE INITIATIVE/PROGRAMME

80. WHAT DIMENSIONS WERE CONSIDERED WITHIN THE INITIATIVE/PROGRAMME AND WHAT IS THEIR IMPORTANCE:	Not relevant/importan t	Relevant, but not considered	Relevant and partly considered	Relevant and considered
SPATIAL DIMENSION (GEOGRAPHIC DIMENSION OF THE INITIATIVE	◯	◯	◯	◯
TIME DIMENSION (CONSIDERS SHORT TO LONG-TERM SCALES RELATIVE TO PURPOSIVE/PRIORITIES	◯	◯	◯	◯
DIMENSIONS OF LIFE (CONSIDERS ALL THE ORGANISMS INVOLVED IN THE DISEASE (E.G. PATHOGENS –TICK VECTORS-HOSTS-ENVIRONMENT	◯	◯	◯	◯
KNOWLEDGE	◯	◯	◯	◯
LEGISLATION	◯	◯	◯	◯
SOCIO-HEALTH (SOCIAL AND ECONOMIC FACTORS/DATA CONSIDERED)	◯	◯	◯	◯
MANAGEMENT (THE LEVEL OF COORDINATION LOCAL-NATIONAL LEVEL)	◯	◯	◯	◯
GOVERNANCE (LOCAL LEADERSHIP AND RESOURCE ALLOCATION)	◯	◯	◯	◯

81.HOW WELL DO THE NUMBER OF DIMENSIONS AND SCALES WITHIN THOSE DIMENSIONS REFLECT AN INTEGRATED APPROACH TO TBD SURVEILLANCE CONTROL IN THE INITIATIVE/PROGRAMME?	0=1 dimension, 1 scale	0.2=2-3 dimensions, 1 scale	0.4=1 dimension, >1 scales	0.6=2-3 dimensions, of which one >1 scales	0.8=2-3 dimensions, few at >1 scales; 1= very comprehensiv e
SPATIAL DIMENSION (LOCAL/REGIONAL, NATIONAL, INTERNATIONAL, GLOBAL)	◯	◯	◯	◯	◯
TIME DIMENSION (SHORT (HOURS), FROM WEEK TO MONTHS, YEARS, DECADES)	◯	◯	◯	◯	◯

DIMENSIONS OF LIFE (GENE, CELLS AND MICROORGANISMS, PATHOGEN HAZARD (FROM TRANSMISSION HOSTS, ENVIRONMENT OR VECTORS); EXPOSURE (TRANSMISSION OR SUSCEPTIBLE HOSTS); IMPACT (SUSCEPTIBLE HOSTS))	○	○	○	○	○
KNOWLEDGE (DATA, TREND/PATTERNS, MODELS, PARADIGMS)	○	○	○	○	○
LEGISLATION (LOCAL/REGIONAL, NATIONAL, EUROPEAN/INTERNATIONAL	○	○	○	○	○
SOCIO-HEALTH (SOCIAL AND ECONOMIC FACTORS/DATA CONSIDERED)	○	○	○	○	○
MANAGEMENT (TASKS, PROJECTS AND STRATEGIES)	○	○	○	○	○
GOVERNANCE (LOCAL/REGIONAL, NATIONAL)	○	○	○	○	○

REVIEWER COMMENTS

....
