# Supplementary material for: Mapping one health surveillance and networks for vector-borne disease prevention and control in Europe – A scoping review
Source: One Health. 2026 Aug 18;23:101545. doi: 10.1016/j.onehlt.2026.101545 (PMC13545381; doi:10.1016/j.onehlt.2026.101545)
Supplement: Supplementary file 2 [file mmc1.docx]

**Table S1: Database search terms**

| **Database** | **Hits** | **Relevant retrieved based on the fist 500** | **Search term** | **Date retrieved** |
| --- | --- | --- | --- | --- |
| **Initial database searches** | | | | |
| Web of Science | 1,012,168* | 148 | tick-borne diseases OR infections OR outbreaks OR epidemics OR zoonotic diseases AND surveillance OR forecasting OR early warning OR informatics OR preparedness OR decision support systems OR risk mapping OR risk assessment OR monitoring AND UK OR England OR Scotland OR Wales OR Northern Ireland AND European Union OR EU | 08-09-2023 |
| Web of Science |  | 114 | (((((((((((((TS=(tick-borne diseases)) OR TS=(infections)) OR TS=(outbreaks)) OR TS=(epidemics)) OR TS=(zoonotic diseases)) AND TS=(surveillance )) OR TS=(forecasting)) OR TS=(early warning)) OR TS=(informatics)) OR TS=(preparedness)) OR TS=(decision support systems)) OR TS=(risk mapping)) OR TS=(risk assessment)) OR TS=(monitoring) OR TS=(virus) AND TS=(one health) OR TS=(ecohealth) OR TS=("global health") AND TS=(tick) AND TS= (vector borne) | 11-09-2023 |
| Web of Science | 259,741 | 53 | tick-borne diseases OR infections OR outbreaks OR epidemics OR zoonotic diseases AND surveillance OR forecasting OR early warning OR informatics OR preparedness OR decision support systems OR risk mapping OR risk assessment OR monitoring AND one health OR ecohealth | 13.09.2023 |
| WoS-4 | 698,514 | 78 | Surveillance OR Monitor OR Monitoring OR Monitored OR Integrate OR Integration OR Integrated OR Integrating AND tick-borne diseases OR infections OR outbreaks OR epidemics OR zoonotic diseases Animal health OR Human health OR Public health OR One Health OR System AND UK OR England OR Wales OR Northern Ireland OR Scotland AND EU OR European Union | 13.09.2023 |
|  |  |  |  |  |
| WoS | 719,123 | 97 | Surveillance OR Monitor OR Monitoring OR Integrate OR Integration OR Integrating OR Preparedness OR Preparing OR Intervention AND tick-borne diseases OR infections OR outbreaks OR epidemics OR vector borne diseases animal health OR Human health OR Public health OR One Health OR System AND UK OR England OR Wales OR Northern Ireland OR Scotland AND EU OR European Union | 18.09.2023 |
| PubMed | 4,017 | 10 | Surveillance OR Monitor OR Monitoring OR Monitored OR Integrate OR Integration OR Integrated OR Integrating AND tick-borne diseases OR infections OR outbreaks OR epidemics OR zoonotic diseases Animal health OR Human health OR Public health OR One Health OR System AND UK OR England OR Wales OR Northern Ireland OR Scotland AND EU OR European Union | 18.09.2023 |
| WoS | 220 | 66 | (Surveillance OR monitor*) AND (“vector borne disease” OR tickborne disease OR “animal health” OR “human health” OR “public health” OR “One Health”) AND (integrate*) AND (system) | 18.09.2023 |
| PubMed | 216 | 10 | (Surveillance OR monitor*) AND ("vector borne disease" OR tickborne disease OR "animal health" OR "human health" OR "public health" OR "One Health") AND (integrate*) AND (system) AND UK | 20.09.2023 |
|  |  | **566** |  |  |
|  |  |  |  |  |
| **Refined and updated searches after iterations** | | | | |
| WoS | 1,168 | **55** | ("vector borne disease" OR tickborne disease OR "One Health") AND (surveillance OR monitor* OR system OR models OR forecast* OR predict*) | 06.11.2023 |
| WoS | 16,704 | **31** | “tick-borne diseases” OR “vector-borne disease” AND “surveillance" OR "forecasting” OR “early warning” OR “informatics” | 07.11.2023 |
| PubMed | 2,775 | **56** | ("vector borne disease" OR tickborne disease OR "One Health") AND (surveillance OR monitor* OR system OR models OR forecast* OR predict*) | 07.11.2023 |
| Google scholar | 980 | **55** | "vector borne disease" OR tickborne disease OR "One Health" AND (surveillance OR monitor* OR system OR models OR forecast* OR predict* | 24.11.2023 |
| Google scholar | 987 | **92** | (Surveillance OR monitor*) AND ("vector borne disease" OR tickborne disease OR "animal health" OR "human health" OR "public health" OR "One Health") AND (integrate*) |  |
|  |  |  |  |  |
| WoS |  | **24** | ("vector borne disease" OR tickborne disease OR "One Health") AND (surveillance OR monitor* OR system OR models OR forecast* OR predict*) | **04.09.2025** |
| WoS |  | **27** | ("vector borne disease" OR tickborne disease OR "One Health") AND (surveillance OR monitor* OR system OR models OR forecast* OR predict*) | **11.09.2025** |
| Google scholar |  | Subset of new WoS retrievals | Surveillance OR monitor*) AND ("vector borne disease" OR tickborne disease OR "animal health" OR "human health" OR "public health" OR "One Health") AND (integrate*) | **26.09.2025** |
